# Preconception, Prenatal and Postnatal PM_2.5_ Exposure and Neurodevelopmental Disorder Risk among Medicaid Recipients

**DOI:** 10.1021/acs.est.6c03766

**Published:** 2026-09-28

**Authors:** Matthew Shupler, Xinye Qiu, Krista F. Huybrechts, Sonia Hernandez-Diaz, Wanyu Huang, Hayon Michelle Choi, Michael Leung, Yaguang Wei, Joel Schwartz, Brent Coull, Evgenia Papatheodorou, Christopher J. McDougle, Antonella Zanobetti, Marc Weisskopf, Stefania Papatheodorou

**Affiliations:** † Department of Epidemiology, 1857 Harvard T.H. Chan School of Public Health, Boston, Massachusetts 02115, United States; ‡ Department of Environmental Health, Harvard T.H. Chan School of Public Health, Boston, Massachusetts 02115, United States; § Cardiovascular Institute, 1859 Beth Israel Deaconess Medical Center, Boston, Massachusetts 02215, United States; ∥ Department of Medicine, 1811 Harvard Medical School, Boston, Massachusetts 02115, United States; ⊥ Division of Pharmacoepidemiology and Pharmacoeconomics, Brigham and Women’s Hospital, Boston, Massachusetts 02115, United States; # Department of Environmental Medicine, Icahn School of Medicine at Mount Sinai, New York, New York 10029, United States; ∇ Department of Biostatistics, Harvard T.H. Chan School of Public Health, Boston, Massachusetts 02115, United States; ○ University Hospital of Ioannina, Department of Psychiatry, Ioannina 455 00, Greece; ◆ Lurie Center for Autism, 2348 Massachusetts General Hospital, Lexington, Massachusetts 02421, United States; ¶ Department of Psychiatry, Harvard Medical School, Boston, Massachusetts 02155, United States; †† Department of Biostatistics and Epidemiology, 574030 Rutgers School of Public Health, Piscataway, New Jersey 08854, United States

**Keywords:** Neurodevelopmental delays, autism spectrum disorder, attention-deficit/hyperactivity disorder, PM_2.5_, prenatal, postnatal

## Abstract

Evidence linking ambient fine particulate matter (PM_2.5_) exposure to childhood neurodevelopmental disorders (NDDs) remains mixed. We conducted a cohort study (2001–2013) of 1,547,244 Medicaid-enrolled mother-child pairs to assess associations between zip-code–level PM_2.5_ exposure during preconception (12-week), prenatal (37-week), and postnatal (3-year) periods and children’s NDD risk. Cox proportional hazards models estimated risks of autism spectrum disorder (ASD), attention-deficit/hyperactivity disorder (ADHD), learning difficulty (LD), speech/language disorder, developmental coordination disorder (DCD), intellectual disability, and behavioral disorder, adjusting for demographics, meteorological characteristics, and area-level socioeconomic indicators. Distributed lag models assessed critical exposure windows and cumulative effects. A 10 μg/m^3^ increase in cumulative prenatal PM_2.5_ exposure was associated with higher ASD risk (Hazard ratio (HR):1.17; 95%CI:1.00,1.37). A critical exposure window for ASD existed during gestational weeks 23–31 (HR:1.09 95%CI:[1.02,1.17]). Cumulative prenatal PM_2.5_ exposure was associated with increased ID risk among Hispanic individuals (HR:1.57 95%CI:[1.02,2.43]) but not among other races/ethnicities. Postnatal PM_2.5_ exposure was associated with increased ADHD risk (HR:1.17 95%CI:[0.94,1.46]) at age 0 years and DCD risk (HR:1.72 95%CI:[0.97,3.07]) at age 1 year. No associations between PM_2.5_ exposure and other NDDs were found. Prenatal and postnatal PM_2.5_ exposure may increase the risk of some NDDs among low-income U.S. children.

## Graphical abstract

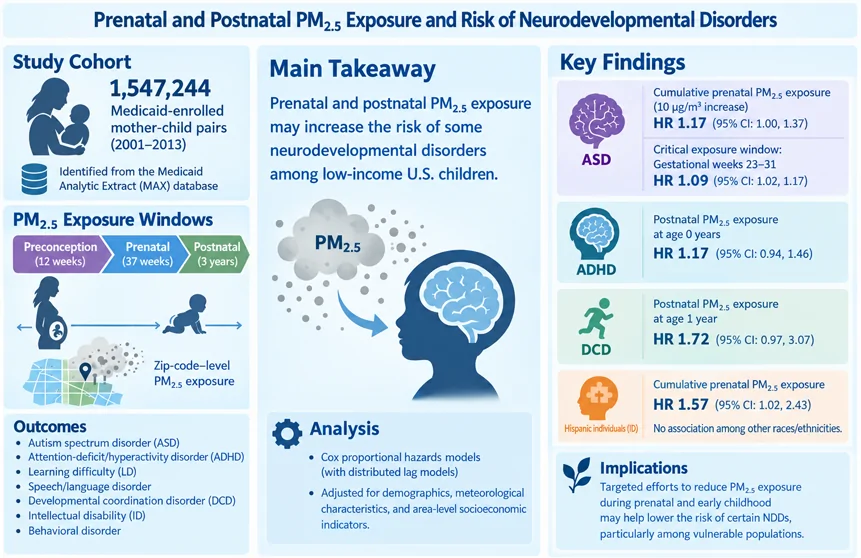

## Introduction

Neurodevelopmental disorders (NDDs) are characterized by delays in social, cognitive, and emotional functioning during early childhood.^1^ They are diagnosed in approximately 10–15% of children in the US,^2^ and are associated with long-term adverse health impacts. NDDs can arise from an interplay of genetic, biological, and environmental influences,^1^ including ambient air pollution exposure. Exposure to fine particulate matter (PM_2.5_), a major component of ambient air pollution, during preconception, prenatal, and early postnatal periods may cause permanent changes in the developing brain and increase the risk of cognitive, behavioral, and motor delays in children.^3^

Existing epidemiological evidence has provided conflicting findings; some studies show that PM_2.5_ exposure is associated with increased NDD risk,^4,5^ and others reporting mixed results.^6,7^ Potential explanations for contrary findings include various methods for ascertaining NDDs, inadequate temporal resolution for PM_2.5_ exposure assessment (e.g., trimester or pregnancy averages), differing degrees of spatial resolution for quantifying PM_2.5_ levels, differences in population characteristics and confounding control, heterogeneity in PM_2.5_ composition and smaller sample sizes for less common NDDs.^8–10^ Studies have also reported differential associations between prenatal and postnatal PM_2.5_ exposure and NDD risk by sex,^11,12^ race/ethnicity,^13,14^ and socioeconomic status (SES).^15^

Although most epidemiological studies have examined prenatal and postnatal exposures, the preconception period may also represent an important window of susceptibility for NDD risk. Environmental exposures before conception can influence reproductive and developmental processes through oxidative stress, inflammation, endocrine disruption, and epigenetic modifications affecting both maternal and paternal gametes.^16,17^ Experimental and epidemiologic evidence suggests that air pollution exposure may alter sperm quality, oocyte competence, DNA methylation patterns, and other molecular pathways that contribute to early embryonic development.^18–20^ Because these biological processes occur before pregnancy, restricting analyses to prenatal and postnatal periods may overlook potentially important exposure windows. Despite growing interest in developmental origins of health and disease, relatively few studies have examined whether preconception PM_2.5_ exposure influences the risk of neurodevelopmental disorders in offspring.

Longitudinal studies assessing early life exposure windows can help delineate the effects of ambient PM_2.5_ exposures on neurodevelopment.^21^ Leveraging a national large population-based cohort of 1.5 million births and high-resolution geospatial air pollution data, this longitudinal study investigates whether PM_2.5_ exposure during preconception, prenatal, and postnatal periods is associated with NDD risk among Medicaid enrollees. We also investigate effect measure modification by sex, race/ethnicity and urbanicity to determine if certain subpopulations may have higher susceptibility to NDD due to PM_2.5_ exposure.

## Methods

We constructed a population-based cohort of pregnant women enrolled in Medicaid from 2001 to 2013. Medicaid is a state and federal health insurance program available to low-income US individuals and covers medical expenses of >40% of births nationally.^22^ Medicaid beneficiary enrollment and healthcare utilization claims were obtained from the Medicaid Analytic Extract (MAX) data set and Transformed Medicaid Statistical Information System Analytic Files (TAF);^23^ cohort creation methods have been described elsewhere.^24^ Pregnant women enrolled in Medicaid are predominantly younger, more racially and ethnically diverse, and economically disadvantaged compared to the general population.

The study population consisted of liveborn children of females aged 12–55 years old enrolled in Medicaid at least three months before the date of their estimated last menstrual period (LMP) to at least one month after delivery. We defined the cohort based on the estimated date of conception (DOC), which was approximated by assuming the DOC fell 2 weeks after the LMP date, assuming a 28-day cycle.

Because cohort entry was defined by estimated conception date, pregnancies were included in our study regardless of gestational duration.^25^ We restricted inclusion to conceptions occurring between January 1, 2001 and December 31, 2013 to additionally ensure complete ascertainment of preconception, prenatal, and three-year postnatal exposure histories within the available PM_2.5_ data set (January 1, 2000–December 31, 2016).

Postnatal follow-up time was calculated as the number of days from a child’s birth until either their enrollment in Medicaid ended, the development of a NDD, death, or study period end, whichever came first, with up to 12 years of maximum follow-up. Children with any chromosomal or genetic condition were excluded.

### Exposure Assessment

Average zip code-level PM_2.5_ concentrations were assigned to each mother based on their residential zip code. The PM_2.5_ measurements were obtained from an ensemble model that estimates spatiotemporally resolved concentrations at a 1 km^2^ grid resolution across the contiguous US from 2000 to 2016.^26^ The model has been applied in other epidemiological studies.^27^ The 1-km^2^ grid–cell daily PM_2.5_ concentration predictions were aggregated at a zip code-level by averaging grid cells whose centroids were inside the polygonal area of the zip code.^27^ For prenatal exposure assessment, residential zip codes recorded at the estimated last menstrual period (LMP) and at delivery were used. If the zip codes differed, we attributed 50% of the pregnancy exposure duration to each zip code, consistent with previous studies.^28^ For postnatal exposure assessment, residential zip codes were updated annually using Medicaid enrollment records, allowing exposure assignment to account for residential mobility during the three-year postnatal follow-up period.

### Outcome Variables

Outcome data from the MAX database included a total of seven NDDs: autism spectrum disorder (ASD), attention-deficit/hyperactivity disorder (ADHD), learning disability, developmental speech or language disorders, developmental coordination disorder (DCD), intellectual disability (ID), and behavioral disorder. Validated claims-based algorithms were used to define each outcome (Table S1).^29^ The algorithms, which have high positive predictive values (range: 82–98%), integrate data from International Classification of Diseases (ICD)-9 claims codes, age at diagnosis and pharmacy dispensing records for relevant medications. The overall trends and frequencies of diagnosed NDD in our cohort were broadly comparable to those reported in U.S. surveillance systems and other studies.^2^ The coding of NDD was thoroughly validated through medical record review; researchers identified positive predictive values generally >90% for all NDD included in this study.^29^

### Covariates

We adjusted for individual and area-level factors, as in prior studies.^28,30^ Individual-level variables included maternal age, maternal race/ethnicity, substance abuse, smoking, alcohol abuse, poor nutrition, folate use during pregnancy, birth year, and season of conception (Table S2). We included race/ethnicity as a covariate as a proxy for life experiences of structural racism.^31^ Area-level SES variables included median household income, median value of owner-occupied housing, percentage of population living below the poverty level, percentage of population with less than high school education, percentage of owner-occupied housing units, and population density^32^ as a proxy for urbanicity (Table S2). Area-level SES data were obtained from the 2000 and 2010 US Census and linearly interpolated. After 2010, annual estimates were obtained from the American Community Survey, which has been used in previous epidemiological studies.^33,34^ We acquired county-level body mass index (BMI) annually from 2000 to 2012 from the Behavioral Risk Factor Surveillance System.^35^ We also adjusted for zip code-level mean Normalized Difference Vegetation Index (NDVI)^36^ as a proxy for greenness exposure, and distance to the nearest hospital as a surrogate measurement for local healthcare access.^37^ The relationship between the exposure, outcome and covariates was mapped in a directed acyclic graph (Figure S1).

### Meteorological Variables

Daily ambient air surface temperatures and relative humidity across the contiguous US were obtained at a 4 km^2^ spatial resolution from a hybrid model^38^ using NASA’s Land Data Assimilation System Phase 2 (NLDAS-2)^39^ and the Parameter-elevation Regressions on Independent Slopes Model (PRISM).^40^ Meteorological data was also aggregated by zip code.

### Statistical Analysis

We estimated hazard ratios (HRs) and 95% confidence intervals (CIs) using stratified Cox proportional hazards models. PM_2.5_ exposure was modeled using distributed lag models (DLMs) within the stratified Cox modeling framework. Separate Cox proportional hazard models were fitted for the preconception/prenatal and postnatal periods.

Variables that violated the proportional hazards assumption were included as stratification factors, allowing baseline hazards to vary across strata. Accordingly, models were stratified by child sex, birth year, race/ethnicity, and county-level Federal Information Processing Systems (FIPS) code. Standard errors were clustered at the zip-code level to account for within-area correlation.

For the preconception and prenatal analyses, weekly PM_2.5_ exposures were modeled across the 12-week preconception period and the first 37 weeks of gestation, respectively. Because DLMs require complete exposure profiles, the primary prenatal DLM analysis was restricted to pregnancies with complete weekly exposure information through 37 weeks of gestation. We selected 37 weeks because most pregnancies reach this gestational age, thereby minimizing selection bias through conditioning on gestational duration while allowing evaluation of late-pregnancy exposure windows. Thus, using 37 weeks in the DLMs ensured that nearly all pregnancies contributed complete exposure information regardless of eventual gestational duration. For postnatal analyses, DLMs were used to evaluate PM_2.5_ exposure during the first three years of life; we examined NDD risk due to annual average PM_2.5_ concentrations to align with the annual resolution of the residential histories provided in the MAX database.

As prenatal and postnatal PM_2.5_ exposures were highly correlated (Spearman correlation coefficients: 0.81–0.88) and failure to account for prenatal exposure may confound estimates of the independent association between postnatal PM_2.5_ exposure and health outcomes,^41^ postnatal models were additionally adjusted for prenatal PM_2.5_ concentrations using a cross-basis function for the main analysis. We present unadjusted postnatal models as a sensitivity analysis. However, preconception/prenatal models were not adjusted for postnatal exposure, as adjustment for postnatal PM_2.5_ concentrations would be controlling for a chronologically later exposure that may lie on the causal pathway and could introduce bias.

We used DLMs to estimate lag-specific and cumulative associations for all (preconception, prenatal and postnatal) exposure windows. We selected the degrees of freedom (df) for the lag-response function by minimizing the Akaike Information Criterion (AIC). PM_2.5_ concentrations were modeled using a linear exposure-response function and 6 df for the lag-response function. Temperature and relative humidity were modeled using linear exposure-response functions and 4 df for the lag-response functions.

Hazard ratios are presented per 10 μg/m^3^ increase in PM_2.5_ concentration. This scaling factor was selected to facilitate comparison with prior air pollution epidemiology studies.^42,43^ Because PM_2.5_ was modeled continuously, the choice of scaling factor affects only the presentation of effect estimates and not model fitting or statistical inference. A reference concentration of 9 μg/m^3^ was selected because it corresponds to the current U.S. National Ambient Air Quality Standard.

### Effect Modification Analyses

As sex, race/ethnicity, and urbanicity^44^ (population density of > or <500 people/zip code) have been shown to modify the association between PM_2.5_ exposure and NDD risk, effect modification was evaluated by these factors. Interaction terms were created between each effect modifier and average PM_2.5_ concentration during the corresponding exposure period. Statistically significant interactions were further explored using stratified analyses.

We additionally conducted region-specific analyses within five U.S. Census regions (Northeast, Southeast, Midwest, Southwest, and West) (Figure S2)^45^ to evaluate geographic heterogeneity in associations, which has been done in other air pollution epidemiology studies.^46^

### Sensitivity Analyses

Because loss of Medicaid enrollment during follow-up could introduce informative censoring, we conducted inverse probability of censoring weighted (IPCW) analyses to evaluate the robustness of our findings to potential selection bias.^47,48^ IPCW analyses were considered sensitivity analyses because they require additional assumptions regarding the censoring process and specification of the censoring model. Similarity between the IPCW and primary analyses would provide reassurance that differential loss to follow-up did not materially influence the results.

Additional sensitivity analyses included: (1) restricting distributed lag models to pregnancies reaching 26 and 30 weeks of gestation to check for the impact of the duration of gestation in our associations,^49^ (2) additional adjustment for ambient NO_2_ concentrations,^50^ which are correlated with PM_2.5_ concentrations and may have independent impacts on NDD risk;^51^ (3) restricting outcome ascertainment to diagnoses occurring after age three years to focus on cases whose etiology may be more strongly driven by environmental factors;^52^ and (4) varying the degrees of freedom used in the exposure-response and lag-response functions to assess the robustness of our findings to model specification. In the sensitivity analysis, NO_2_ was included as a continuous variable using the average concentration during the corresponding exposure period and was entered linearly into all models.

Our interpretation of the results focused on the strength of the adjusted HR and its precision (width of 95% CI), rather than only on statistical significance at an α <0.05 level.^51^ However, statistical significance was relied on when identifying potential susceptible windows of exposure using the DLMs. All analyses were conducted in R version 4.1.1.^53^ The study protocol was approved by Harvard TH Chan School of Public Health (IRB23–1090), Mass General Brigham (2022P002615), and Rutgers School of Public Health (2024000897). As this study involved secondary analysis of previously collected, deidentified Medicaid claims data and no direct contact with participants, the requirement for informed consent was waived.

## Results

After excluding participants with missing data, the final analytic sample included 1,547,244 mother-child pairs (Figure S3), nearly all of which were singleton births (*n* = 1,526,817; 99%). The most common NDD were developmental speech or language disorder (*n* = 73,911), ADHD (*n* = 67,338), behavioral disorder (*n* = 47,671) and ASD (*n* = 12,942). Among children diagnosed with ASD, 38.7% also had a diagnosis of ADHD. The median age of diagnosis for ASD, ADHD and any NDD was 3.7 years (inner quartile range (IQR): 3.0), 6.0 years (IQR: 2.4) and 5.0 years (IQR: 3.7), respectively. The median total follow-up time for children diagnosed with any NDD was 7.4 years (IQR: 5.2). PM_2.5_ concentrations gradually declined during the study period (Figure S4), with a median weekly prenatal exposure of 11.26 μg/m^3^ (IQR: 9.33–13.17) and median annual postnatal exposure of 10.49 μg/m^3^ (IQR: 8.90–12.27). The proportion of White children was higher in the lower exposure (below median) group, while Black/African American and Hispanic/Latino children were more prevalent in the higher exposure (above/at median) group (Table 1). Substance use indicators (smoking, alcohol, drug use) were more common in the lower PM_2.5_ exposure group. Median household income and educational attainment were lower in higher PM_2.5_ exposure areas. PM_2.5_ concentrations during the preconception, prenatal and postnatal exposure periods were highly correlated (Spearman correlation coefficients: 0.81–0.88).

**Table 1:** Cohort Characteristics Stratified by Median Preconception/Prenatal and Postnatal PM_2.5_ Exposure (*n* = 1,547,244)^a^

		Preconception/Prenatal PM_2.5_ exposure		Postnatal PM_2.5_ exposure	
	Total	Below median (11.26 μg/m^3^)	Above/at median (11.26 μg/m^3^)	Below median (10.49 μg/m^3^)	Above/at median (10.49 μg/m^3^)
**Individual variables**					
Sex of child (Male) [*n* (%)]	781,529 (50.5)	391,458 (50.7)	390,071 (50.3)	391,374 (50.6)	390,155 (50.4)
Maternal race/ethnicity [*n* (%)]					
White	642,592 (41.5)	361,151 (46.6)	281,441 (36.3)	365,548 (47.3)	277,044 (35.8)
Black/African-American	502,273 (32.4)	210,007 (27.1)	292,266 (37.8)	209,267 (27.0)	293,006 (37.8)
Asian/Other Pacific	50,193 (3.2)	28,203 (3.6)	21,990 (2.8)	27,812 (3.6)	22,381 (2.9)
Hispanic/Latino	300,551 (19.4)	137,214 (17.7)	163,337 (21.1)	133,557 (17.3)	166,994 (21.6)
Unknown	51,635 (3.3)	37,047 (4.8)	14,588 (1.9)	37,427 (4.8)	14,208 (1.8)
Maternal age at delivery [mean (sd)]	24.5 (5.9)	24.1 (5.8)	25.0 (5.9)	25.0 (5.9)	24.1 (5.8)
< 18	134,610 (8.7)	58,595 (7.4)	75,527(10)	46,246 (7.1)	87,876 (9.8)
18–45	1,411,534 (91.3)	733,784 (92.6)	679,760 (90.0)	603,096 (92.8)	810,448 (90.2)
> 45	1,100 (0.0)	366 (0.)	265 (0.0)	299 (0.0)	332 (0.0)
Low birth weight [*n* (%)]	116,043 (7.5)	54,022 (7.1)	62,021 (8.0)	55,011 (7.1)	61,032 (7.8)
Gestational diabetes [*n* (%)]	137,704 (8.9)	77,663 (9.8)	60,467 (8.0)	64,980 (10.0)	73,150 (8.1)
Gestational hypertension [*n* (%)]	55,701 (3.6)	30,567 (3.9)	24,721 (3.3)	25,198 (3.9)	30,090 (3.3)
Substance abuse [*n* (%)]	20,809 (1.3)	12,674 (1.6)	8,135 (1.1)	13,012 (1.7)	7,797 (1.0)
Smoking [*n* (%)]	30,942 (2.0)	19,486 (2.5)	11,456 (1.5)	19,801 (2.6)	11,141 (1.4)
Alcohol abuse [*n* (%)]	7,913 (0.5)	4,986 (0.6)	2,927 (0.4)	5,035 (0.6)	2,878 (0.4)
Poor nutrition [*n* (%)]	6,473 (0.4)	4,609 (0.6)	1,864 (0.2)	4,697 (0.6)	1,776 (0.2)
Folate	55,791 (3.6)	29,964 (3.9)	25,827 (3.4)	30,611 (4.0)	25,180 (3.3)
**Area-level variables**					
Median household income (thousands ($)) [mean (sd)]	41.9 (15.0)	44.6 (15.9)	39.3 (13.7)	45.0 (15.9)	38.9 (13.4)
Owner-occupied housing value [mean (sd)]	180.8 (142.4)	194.3 (151.7)	167.9 (131.6)	202.3 (156.4)	159.6 (123.5)
Owner-occupied housing units [% (sd)]	57.7 (19.3)	60.2 (18.7)	55.3 (19.6)	59.5 (19.2)	55.9 (19.3)
Above 65 years and did not complete high school [% (sd)]	35.8 (15.6)	31.2 (14.6)	40.2 (15.3)	30.8 (14.4)	40.6 (15.3)
Above 65 years and living below the poverty level [% (sd)]	13.7 (8.5)	12.9 (8.5)	14.5 (8.4)	13.0 (8.6)	14.4 (8.3)
Population Density (people/km^2^) [mean (sd)]	2,670 (5,530)	2,382 (5,457)	2,959 (5,587)	2,616 (5,794)	2,724 (5,252)
BMI [mean (sd)]	27.7 (1.4)	27.8 (1.6)	27.7 (1.1)	27.8 (1.6)	27.7 (1.1)
Nearest hospital (km) [mean (sd)]	5.5 (6.9)	6.4 (7.9)	4.7 (5.5)	6.3 (7.9)	4.8 (5.6)

a sd = standard deviation.

When stratifying sociodemographic factors by NDD diagnosis, we found a higher diagnostic chance among males and White children compared with Black and Hispanic participants (Table S3).

### Preconception and Prenatal Exposures

In fully adjusted models, cumulative (37-week) prenatal PM_2.5_ exposure was associated with increased ASD risk (HR: 1.17, 95%CI: [1.00, 1.37]) (Figure 1). The exposure-response curve showed increased ASD risk at higher levels of preconception/prenatal PM_2.5_ exposure (Figure S5). Additionally, a negative association was found between prenatal PM_2.5_ exposure and ADHD risk (HR: 0.90, 95%CI: [0.83, 0.99]) (Figure 1). No associations were found between cumulative preconception or prenatal PM_2.5_ exposure and any other NDD among the overall study population.

**Figure 1:**
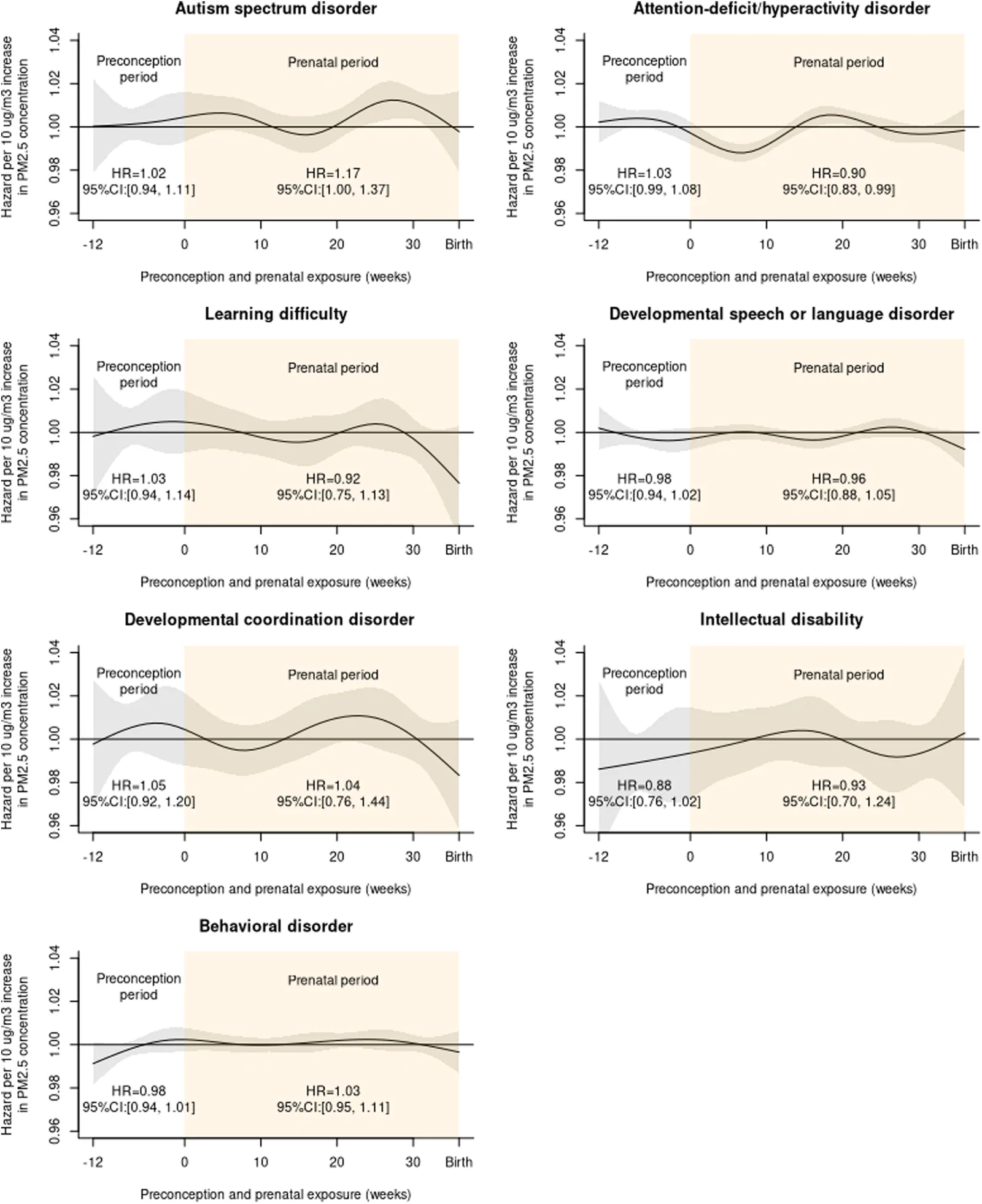
Lag-response relationship between PM_2.5_ concentration (10 μg/m^3^ increase) and NDD during preconception and prenatal period (adjusted for temperature, relative humidity, individual and area-level covariates). Hazard ratios [95% CI] for association between preconception and prenatal PM_2.5_ exposure and neurodevelopmental delays at the cumulative lag (12 weeks for preconception and 37 weeks for prenatal).

### Preconception and Prenatal Critical Exposure Windows

A critical exposure window for increased ASD risk occurred during weeks 23–31 of gestation, with a 10 μg/m^3^ increase in cumulative PM_2.5_ exposure associated with 1.09 times (95%CI:[1.02, 1.17]) the risk of ASD (Figure 1). Additionally, a 10 μg/m^3^ increase in PM_2.5_ levels at 16–22 weeks gestation was associated with 1.03 times (95%CI:[1.01, 1.06]) the risk of ADHD. Prenatal PM_2.5_ exposure at gestational weeks 1–15 was associated with reduced ADHD risk (0.90 95%CI:[0.85,0.94]).

### Postnatal Exposures

After adjustment for prenatal/preconception PM_2.5_ exposure, postnatal PM_2.5_ exposure was associated with higher DCD risk (1.81 95%CI:[0.94, 3.47]) (Figure 2). The exposure-response curve showed increased DCD risk at higher levels of postnatal PM_2.5_ exposure (Figure S6). No associations were found between cumulative postnatal PM_2.5_ concentrations and risk of other NDD. In models without adjustment for preconception/prenatal PM_2.5_ exposure, there were no substantial changes in the HRs for postnatal PM_2.5_ exposure (Table S4).

**Figure 2:**
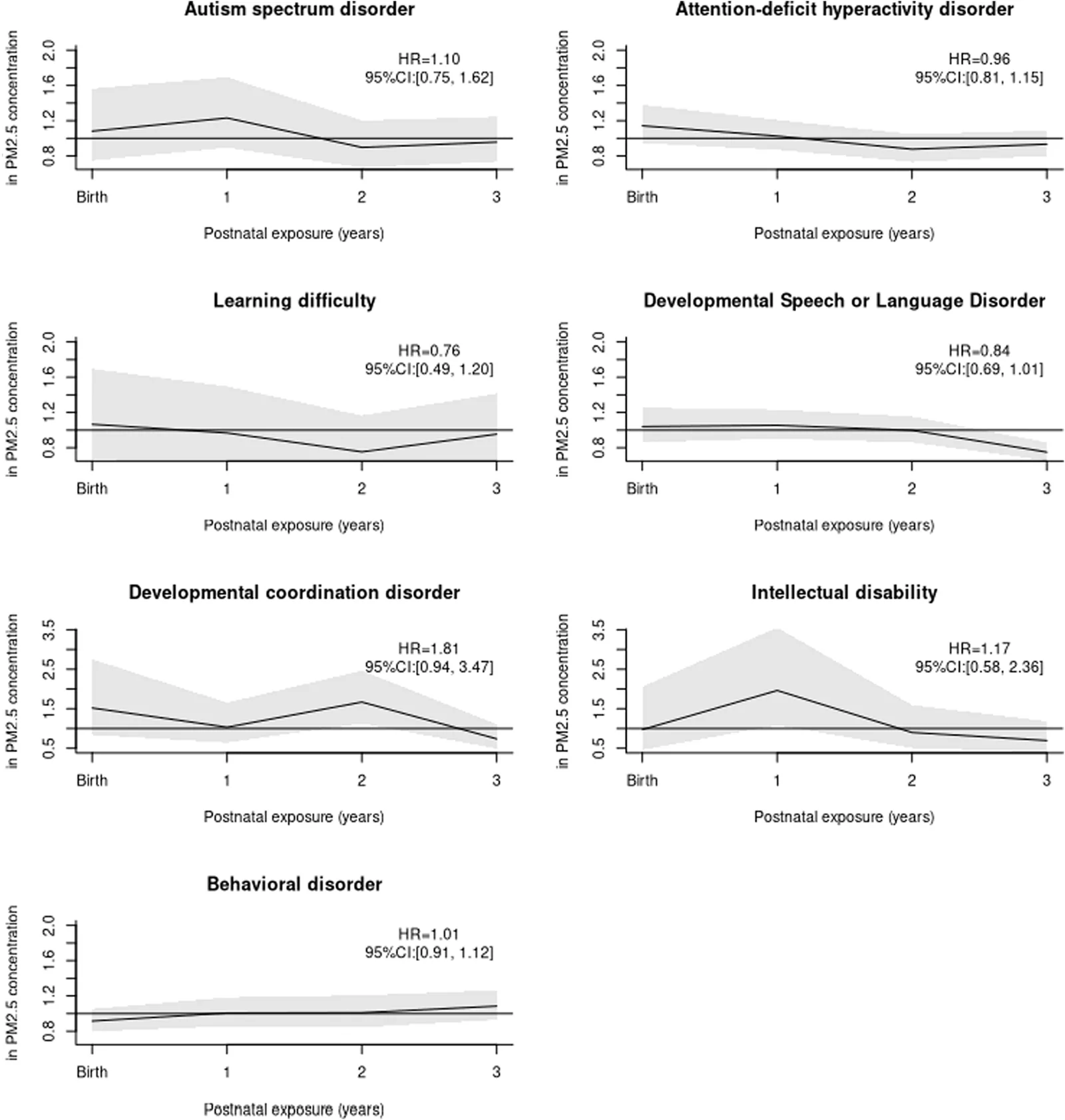
Lag-response relationship between PM_2.5_ concentration (10 μg/m^3^ increase) and NDD during postnatal period (adjusted for temperature, relative humidity, individual and area-level covariates, and preconception/prenatal PM_2.5_ levels). Hazard ratios [95% CI] for association between postnatal PM_2.5_ exposure and neurodevelopmental delays at the cumulative lag (0–3 years).

### Susceptible Windows in the Postnatal Period

Postnatal PM_2.5_ levels at age 0 years was associated with ADHD risk (1.17 95%CI:[0.94, 1.46]) (Table S5; Figure 2). Postnatal PM_2.5_ exposure at age 1 year was associated with risk of DCD (1.72 95%CI:[0.97, 3.07]). There was suggestive evidence that postnatal PM_2.5_ levels at age 1 (1.91 95%CI:[0.84, 4.36]) and 2 years (1.76 95%CI:[0.88, 3.54]) were associated with ID risk.

### Effect Modification during the Prenatal Exposure Period

We identified that race/ethnicity was an effect modifier in the association between prenatal PM_2.5_ exposure and ADHD risk (*p*_interaction_ < 0.001) (Figure 3). The association between cumulative prenatal PM_2.5_ exposure and ADHD risk was also higher among Hispanic participants (1.12 95%CI: [0.94, 1.33]), compared with Black (0.89 95%CI:[0.75, 1.04]) and White (0.79 95%CI:[0.70, 0.89]) individuals. Additionally, there was an association between prenatal PM_2.5_ exposure and ID risk among Hispanic enrollees (1.57 95%CI:[1.02, 2.43]), but not White individuals (0.94 95%CI:[0.61, 1.48]). The sex of the child (Table S6) and urbanicity (Table S7) did not modify the association between preconception/prenatal exposure and NDD risk. When stratifying models by US region, there were not substantial differences in the association between cumulative preconception and prenatal PM_2.5_ exposure with NDD risk (Table S8).

**Figure 3:**
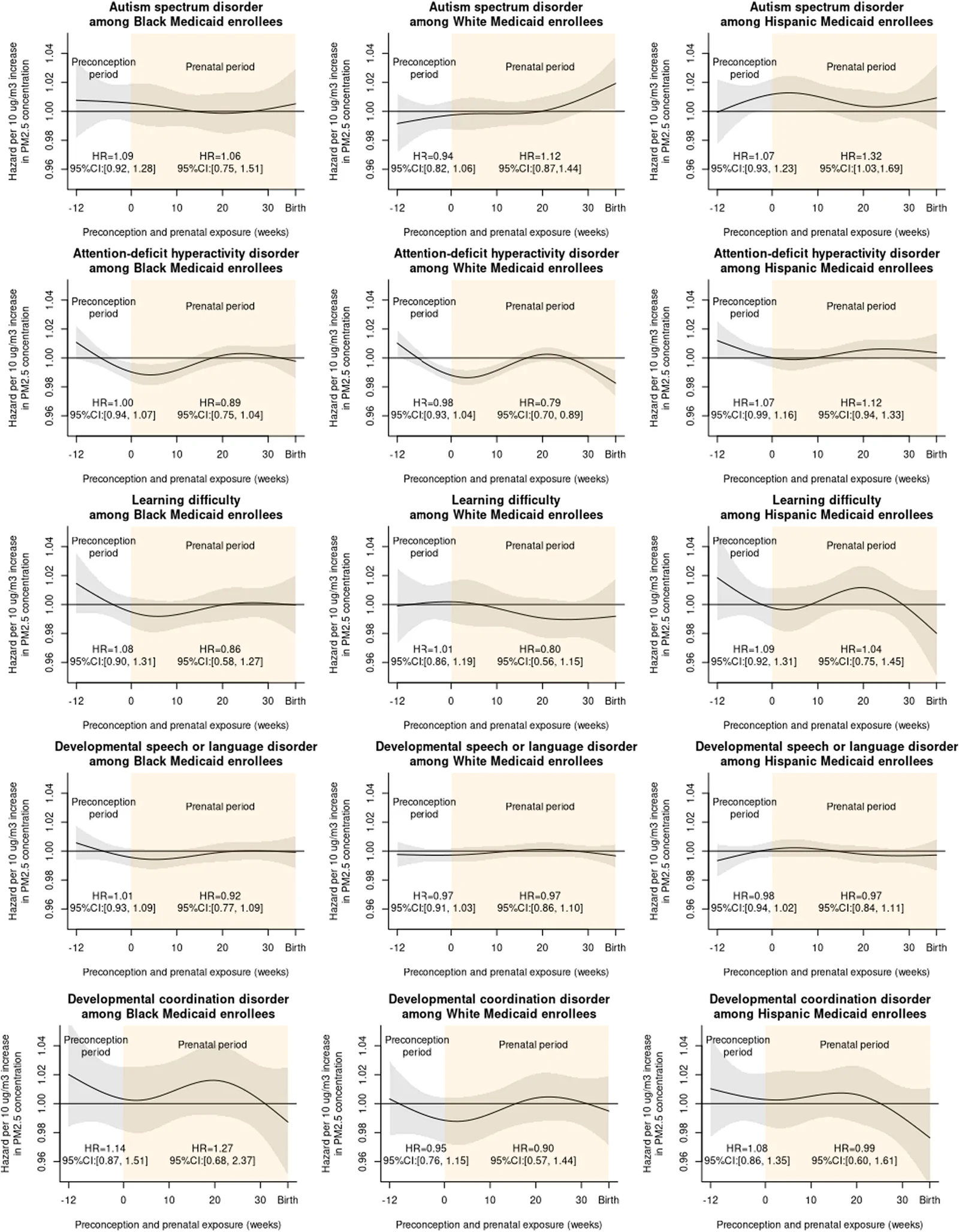
Lag-response relationship between PM_2.5_ concentration (10 μg/m^3^ increase) and NDD during preconception and prenatal period (adjusted for temperature, relative humidity, individual and area-level covariates, and preconception/prenatal PM_2.5_ levels) stratified by race/ethnicity. Hazard ratios [95% CI] for association between preconception and prenatal PM_2.5_ exposure and neurodevelopmental delays at cumulative lag (12 weeks for preconception and 37 weeks for prenatal).

### Effect Modification during the Postnatal Exposure Period

Participants’ race/ethnicity modified the association between postnatal PM_2.5_ exposure and ADHD (*p*_interaction_ = 0.001) risk (Figure 4). Specifically, Black (1.10 95%CI:[0.79, 1.53]) and Hispanic (1.28 95%CI:[0.91, 1.81]) enrollees had an elevated risk of ADHD due to PM_2.5_ exposure, while White participants did not (0.90 95%CI:[0.69, 1.16]). The child’s sex (Table S9) and urbanicity (Table S10) did not modify the association between postnatal PM_2.5_ exposure and risk of any NDD.

**Figure 4:**
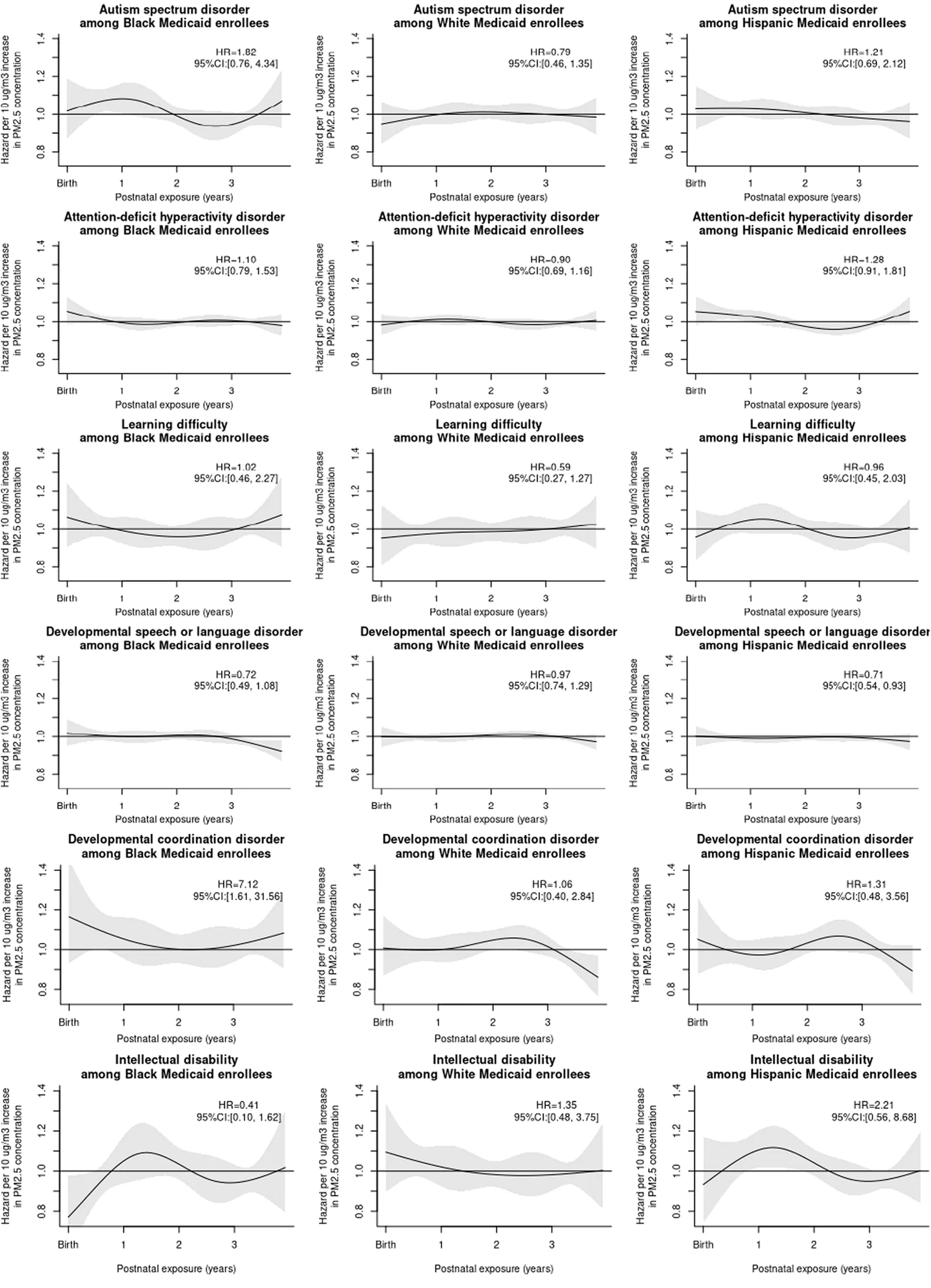
Lag-response relationship between PM_2.5_ concentration (10 μg/m^3^ increase) and NDD during postnatal period (adjusted for temperature, relative humidity, individual and area-level covariates, and preconception/prenatal PM_2.5_ levels) stratified by race/ethnicity. Hazard ratios [95% CI] for association between postnatal PM_2.5_ exposure and neurodevelopmental delays at cumulative lag (0–3 years).

### Sensitivity Analyses

In a sensitivity analysis that included models containing shorter maximum lags (26- and 30-week) to account for different gestation durations, HRs (Table S11) and the shape of the lag-response curves (Figure S7) were similar. When including only children with an NDD diagnosis at age 3 or later (less severe cases), the HRs were similar to those generated from the main models (Table S12). When adjusting for average ambient NO_2_ concentrations, minimal changes in the HRs occurred (Table S13). Finally, accounting for censoring weights did not alter the findings (Table S14). Results from sensitivity analyses using alternative degrees of freedom for the PM_2.5_ exposure–response and lag–response functions were highly consistent with those from the primary models. Across specifications using 3 and 4 degrees of freedom in both dimensions, the overall shape of the exposure–lag–response surfaces remained similar, and the timing of gestational windows associated with increased ASD risk was preserved.

## Discussion

In this national study of more than 1.5 million children, we found that prenatal PM_2.5_ exposure was associated with increased ASD risk, with a sensitive window of exposure identified during weeks 23–31. We also observed associations between prenatal PM_2.5_ exposure during gestational weeks 16–22 and increased ADHD risk, as well as associations between first-year postnatal PM_2.5_ exposure and ADHD risk. Additionally, postnatal PM_2.5_ exposure during the second year of life was associated with an increased risk of DCD. These findings support the hypothesis that exposure to ambient air pollution during specific developmental periods may contribute to the etiology of neurodevelopmental disorders.

Our findings related to ASD risk are generally consistent with previous epidemiologic studies.^6,8–10,54–56^ Previous studies have reported that late pregnancy PM_2.5_ ^57^ and PM_10_ ^58^ exposures had the strongest association with increased ASD risk, and a large body of literature has linked both prenatal and postnatal PM_2.5_ exposure to ASD. Similarly, another study identified gestational weeks 16–22 as a susceptible prenatal window for ADHD, although it reported a broader postnatal susceptible period spanning ages 1–3 years.^59^ Two studies in Taiwan^60^ and China^61^ also found significant associations between first-year PM_2.5_ exposures and ADHD risk.

Beyond ASD and ADHD, we identified a potential association between postnatal PM_2.5_ exposure and the development of DCD, with a possible susceptible exposure window occurring during age 2 years. While previous evidence regarding associations between PM_2.5_ exposure and DCD risk remains limited, a recent study in China found that average PM_2_._5_ exposure during ages 0–3 years was associated with poorer motor performance.^62^

We additionally observed an apparent protective association between PM_2.5_ exposure during gestational weeks 1–15 and ADHD risk. This finding may be reflective of residual confounding or live birth bias, whereby the selective survival between conception and birth alters the distribution of prenatal PM_2.5_ exposures among live-born children.^63^ Such bias could also attenuate associations between prenatal PM_2.5_ exposure and ASD risk.

Several biological mechanisms may explain these findings. PM_2.5_ exposure has been associated with systemic inflammation, oxidative stress, immune dysregulation, placental dysfunction, and neuroinflammation, all of which may disrupt critical neurodevelopmental processes.^64^ Experimental evidence suggests that particulate matter can impair placental function, reduce oxygen and nutrient transport to the fetus, and alter inflammatory signaling pathways involved in fetal brain development.^3^ PM_2.5_ may also cross the blood-brain barrier or induce peripheral inflammatory responses that activate microglia and alter neuronal differentiation, migration, synaptogenesis, and connectivity.^64,65^

The susceptible window for ASD identified during gestational weeks 23–31 coincides with important stages of white matter development. Myelination begins during the late second trimester, and disruption of oligodendrocyte maturation during this period may impair neural circuit formation. Consistent with this hypothesis, post-mortem studies of individuals with ASD have reported deficits in myelination relative to neurotypical controls. In addition, gestational weeks 29–31 correspond to a period of rapid increases in thalamocortical functional connectivity, which plays a central role in shaping cortical organization.^66^ Disruption of these processes through PM_2.5_-induced inflammation and oxidative stress may contribute to ASD pathophysiology.^67,68^ The observed postnatal associations are also biologically plausible because infancy and early childhood represent periods of rapid brain growth and maturation during which environmental insults may have lasting neurodevelopmental consequences.

ASD and ADHD risk due to postnatal PM_2.5_ exposure was higher among Black and Hispanic Medicaid enrollees compared with White individuals. Additionally, the risk of ASD, ADHD and ID due to prenatal PM_2.5_ exposure was higher among Hispanic Medicaid recipients compared with Black and White individuals. Other air pollution epidemiological studies have reported higher risk among racial/ethnic minorities,^27,54^ with potential explanations being that Black and Hispanic communities in the US are generally closer to freeways and industrial facilities^69^ and face negative psychosocial stressors related to systemic racism.^70^ These social determinants may exacerbate the effects of PM_2.5_ exposure on NDD risk through neuroendocrine, vascular, or immune mechanisms involved in the body’s stress response.^71^

Additionally, race can serve as a proxy for structurally patterned pathways (e.g., segregation, differential access to services, diagnostic practices) that impact who is exposed to higher PM_2.5_ levels, and who is evaluated and diagnosed as having an NDD. Multiple studies have found that children with ASD indicators from racial and ethnic minority groups are less likely to have a clinical diagnosis of ASD,^72,73^ including those specifically in the Medicaid-eligible population.^74^

As preconception, prenatal, and postnatal PM_2.5_ exposure was protective for intellectual disability among Black Medicaid enrollees but a risk factor among Hispanic enrollees, methodological limitations such as residual confounding or differential exposure misclassification may also be at play. Intellectual disability diagnoses are also strongly influenced by the timing and intensity of clinical evaluation, which may differ systematically across racial/ethnic groups due to variation in access to developmental screening, referral pathways, and diagnostic substitution with other neurodevelopmental or behavioral diagnoses. Thus, subgroup-specific biases in claims-based outcome ascertainment that vary across exposure windows can result. Moreover, PM_2.5_ exposure may act as a proxy for other environmental and structural contexts across racial/ethnic groups, such correlated neighborhood factors, healthcare utilization patterns, and survival or follow-up processes within Medicaid, rather than biological effects of PM_2.5_ itself. Further epidemiological research would be useful to confirm whether the protective effect in this study remains or may be due to these other factors.

In this national US population-based cohort with more than 1.5 million Medicaid enrollees, we quantified the association between PM_2.5_ exposure and risk of several neurodevelopmental disorders for which there is limited research, including developmental coordination disorder, developmental speech or language disorder and intellectual disability. To our knowledge, this is one of the first national environmental pregnancy studies to evaluate the association between PM_2.5_ concentrations and NDD risk among Medicaid recipients, an underrepresented and ethnically diverse population. We were able to examine effect modification by several sociodemographic factors, which have been less investigated due to a more homogeneous study population.^6^

While genetic susceptibility is an important determinant of neurodevelopmental disorder risk and may modify vulnerability to environmental exposures, claims data from Medicaid does not contain information on genetic risk of developing an NDD. However, genetic factors are unlikely to confound associations between ambient PM_2.5_ exposure and NDD risk because they do not independently determine air pollution exposure once structural and socioeconomic factors are accounted for. We therefore interpret unmeasured genetic risk primarily as a source of effect modification rather than confounding and acknowledge this as a limitation our study.

Moreover, claims data do not allow differentiation between formal diagnostic criteria, symptom overlap, or diagnostic sequencing based on standardized clinical assessments, and should be interpreted accordingly. Likewise, residual confounding by unmeasured familial and social influences cannot be excluded.

Our PM_2.5_ exposures were derived from a validated ensemble model with high spatiotemporal resolution, thereby reducing potential exposure misclassification from studies relying on more sparse monitoring networks.^75,76^ Outcome misclassification was reduced as the NDD definitions were validated with high predictive values; outcome misclassification would be nondifferential and bias toward a null finding. We undertook multiple sensitivity analyses to ensure that biases (e.g., informative censoring, outcome misclassification) did not affect our results.

We were unable to account for residential mobility at a smaller temporal scale than annually after birth because residential zip code was provided on a yearly basis in the MAX database. Consequently, postnatal exposures were assigned using zip code at birth and in every year of follow-up, which may have resulted in nondifferential exposure misclassification. However, by accounting for some residential mobility during pregnancy and annually during the postnatal period, exposure misclassification was lower than previous studies that have relied on a single residential location throughout the follow-up period.

Although the ability to detect susceptible exposure windows is a strength of using DLMs, there were many critical windows examined, which carries a higher risk of identifying a false positive association due to larger number of comparisons. However, the susceptible windows we identified for more commonly studied outcomes, such as ASD^57^ and ADHD,^59^ have been acknowledged in other studies. Because distributed lag models require complete exposure histories, our main analyses were restricted to pregnancies reaching 37 weeks of gestation. Although this restriction may introduce selection bias if gestational age lies on the causal pathway between PM_2.5_ exposure and neurodevelopmental outcome risk, our results were consistent in sensitivity analyses using shorter lag windows that reduced conditioning on gestational duration.

While we adjusted our models for concentrations of other air pollutants, such as NO_2_, there are additional pollutants, including sulfur dioxide (SO_2_), that may also be associated with the risk of adverse neurodevelopmental outcomes. We were unable to include this pollutant in our models due to the unavailability of a national SO_2_ exposure model with adequate spatial and temporal resolution.

As individual residential addresses or geographic coordinates were not available in the MAX database, exposure assignment using population-weighted residential locations within zip codes was not possible. Exposure misclassification may exist when assigning zip code-level PM_2.5_ exposures because residential zip codes do not perfectly capture individual-level exposure patterns, particularly in geographically large zip codes.^77^ Moreover, because ambient PM_2.5_ concentrations can be strongly correlated with neighborhood socioeconomic disadvantage, residential segregation, and structural inequities, it can be difficult to disentangle the independent health effects of PM_2.5_ exposure from its role as a proxy for underlying socioeconomic conditions when assessing associations with health outcomes.^78^

The PM_2.5_ exposures were estimated using a validated 1-km spatial-resolution ensemble prediction model and then aggregated to zip codes, providing substantially greater spatial resolution than county-level or monitor-based exposure assignment. Additionally, the validated ensemble prediction model used in our study has demonstrated strong predictive performance across both urban (cross-validated *R* ^2^ ≈ 0.89) and rural regions (cross-validated *R* ^2^ ≈ 0.77) of the US.^26^ However, a previous simulation analysis showed that within-zip code clustering, resulting in individuals residing within the same zip code receiving identical exposure estimates, can result in underestimated standard errors and biased effect estimates.^79^ Because this measurement error is not expected to be associated with neurodevelopmental disorder diagnoses, it would likely attenuate true associations. Furthermore, the same simulation analysis found that DLMs using low-spatial-resolution exposure data may be prone to bias amplification and variance inflation.^79^ Thus, although exposure assignment was independent of subsequent neurodevelopmental disorder diagnosis and resulting measurement error is expected to be predominantly nondifferential, the direction and magnitude of bias cannot be definitively predicted. Cumulative associations and, particularly, the identification of specific sensitive exposure windows should therefore be interpreted cautiously.

Finally, residential address-level exposure estimates may not fully represent personal exposure because individuals spend substantial time away from their homes. A recent study comparing zip code-level and residential address-level PM_2_._5_ exposure assignment reported nearly identical associations with incident cardiovascular disease, suggesting that zip code-level exposure assignment may provide effect estimates comparable to those obtained using residential address-level exposures.^80^

## Implications

Our study contributes to growing evidence that prenatal PM_2.5_ exposure is a plausible risk factor for ASD and ID, and that postnatal PM_2.5_ exposure may increase ADHD and DCD risk. As several NDD, such as ASD and ADHD, are being increasingly diagnosed in the US,^2,3^ it is critical to continue enacting policies that lower PM_2.5_ exposures to protect early child development.

## Supplementary Material



## Data Availability

The environmental exposure data used in this study is publicly available online at the National Aeronautics and Space Administration Socioeconomic Data and Applications Center (https://sedac.ciesin.columbia.edu/data/collection/aqdh/sets/browse). The outcome data obtained from Medicaid cannot be shared for reasons of confidentiality.

## Notes

Definition of neurodevelopmental disorders and covariates, cohort characteristics, hazard ratios for individual years of follow-up period, models stratified by sex, urbanicity and US region, models with inverse probability weights, direct acyclic graph, sample flowchart, exposure-response relationships (PDF)
