# Supplementary material for: Preconception, Prenatal and Postnatal PM2.5 Exposure and Neurodevelopmental Disorder Risk among Medicaid Recipients
Source: Environ Sci Technol. 2026 Sep 28;60(40):28312–24. doi: 10.1021/acs.est.6c03766 (PMC13674074; doi:10.1021/acs.est.6c03766)

Supplementary Content

Preconception, prenatal and postnatal $PM_{2.5}$ exposure and neurodevelopmental disorder risk among Medicaid recipients

Matthew Shupler, PhD[a], Xinye Qiu, PhD[b,c,d], Krista F. Huybrechts, PhD[e], Sonia Hernandez-Diaz, MD, DrPH[a], Wanyu Huang, PhD[b], Hayon Michelle Choi, PhD[b], Michael Leung, PhD[b], Yaguang Wei, PhD[f], Joel Schwartz, PhD.[a,b], Brent Coull, PhD[g], Evgenia Papatheodorou, MD[h], Christopher J McDougle, MD[i,j], Antonella Zanobetti, PhD[b], Marc Weisskopf, PhD, ScD,[a,b], Stefania Papatheodorou, MD, PhD[k]

a. Department of Epidemiology, Harvard T.H. Chan School of Public Health, Boston, Massachusetts, USA
b. Department of Environmental Health, Harvard T.H. Chan School of Public Health, Boston, Massachusetts, USA
c. Cardiovascular Institute, Beth Israel Deaconess Medical Center, Boston, MA, USA
d. Department of Medicine, Harvard Medical School, Boston, MA, USA.
e. Division of Pharmacoepidemiology and Pharmacoeconomics, Brigham and Women's Hospital, Boston, Massachusetts, USA
f. Department of Environmental Medicine, Icahn School of Medicine at Mount Sinai, New York, New York, USA
g. Department of Biostatistics, Harvard T.H. Chan School of Public Health, Boston, Massachusetts, USA
h. University Hospital of Ioannina, Department of Psychiatry, Ioannina, Greece
i. Lurie Center for Autism, Massachusetts General Hospital, Lexington, MA, USA
j. Department of Psychiatry, Harvard Medical School, Boston, MA, USA
k. Department of Biostatistics and Epidemiology, Rutgers School of Public Health, Piscataway, New Jersey, USA

Table S1. Definition of neurodevelopmental disorders
Table S2. Definition of select individual and area-level covariates
Table S3. Cohort characteristics stratified by neurodevelopmental disease diagnosis (n=1,547,244)
Table S4. Hazard ratio for the association between average annual postnatal $PM_{2.5}$ exposure (10 μg/m^3 increase) and risk of neurodevelopmental disorders at cumulative lag (0-3 years), controlling for average prenatal and preconception exposure
Table S5. Hazard ratio [95% CI] for cumulative association between $PM_{2.5}$ exposure and risk of neurodevelopmental disorders during each year of follow up in the postnatal period
Table S6. Hazard ratios [95% CI] for association between average weekly preconception and prenatal $PM_{2.5}$ exposure (10 μg/m^3 increase) and risk of neurodevelopmental disorders at the cumulative lag (12 weeks preconception and 37 weeks prenatal) from models stratified by sex.
Table S7. Hazard ratios [95% CI] for association between average weekly preconception and prenatal $PM_{2.5}$ exposure (10 μg/m^3 increase) and risk of neurodevelopmental disorders at the cumulative lag from models stratified by urbanicity (> or < 500 residents per zip code)
Table S8. Hazard ratios [95% CI] for association between average weekly preconception and prenatal $PM_{2.5}$ exposure (10 μg/m^3 increase) and risk of neurodevelopmental disorders at the cumulative lag from models stratified by US region
Table S9. Hazard ratios [95% CI] for association between average annual postnatal $PM_{2.5}$ exposure (10 μg/m^3 increase) and risk of neurodevelopmental disorders (controlling for prenatal and preconception $PM_{2.5}$ exposure) at cumulative lag (0-3 years) from models stratified by sex
Table S10. Hazard ratios [95% CI] for association between average annual postnatal $PM_{2.5}$ exposure (10 μg/m^3 increase) and risk of neurodevelopmental disorders (controlling for prenatal and preconception $PM_{2.5}$ exposure) at cumulative lag (0-3 years) from models stratified by urbanicity (> or < 500 residents per zip code)
Table S11. Cumulative hazard ratios [95% CI] for association between average weekly preconception and prenatal $PM_{2.5}$ exposure (10 μg/m^3 increase) and risk of neurodevelopmental disorders (12 weeks preconception and 37 weeks prenatal) at different lag times

Table S12. Hazard ratios [95% CI] for the association between average weekly preconception and prenatal $PM_{2.5}$ exposure (10 μg/m^3 increase) and risk of neurodevelopmental disorders among children diagnosed at age 3 or later
Table S13. Hazard ratios [95% CI] for the association between average weekly preconception and prenatal $PM_{2.5}$ exposure (10 μg/m^3 increase) and risk of neurodevelopmental disorders at the cumulative lag (12 weeks preconception and 37 weeks prenatal and 0-3 years postnatal), controlling for average cumulative NO_2 concentration
Table S14. Hazard ratios [95% CI] for association between average weekly preconception and prenatal $PM_{2.5}$ exposure (10 μg/m^3 increase) and risk of neurodevelopmental disorders at the cumulative lag (12 weeks preconception and 37 weeks prenatal and 0-3 years postnatal), accounting for inverse probability of censoring weights

Figure S1. Direct acyclic graph of the association between prenatal $PM_{2.5}$ exposure and risk of autism spectrum disorder (ASD)
Figure S2. Map of five regions of the United States according to the US Census
Figure S3. Flowchart describing arrival to the final analytic sample in the Medicaid Analytic eXtract database
Figure S4. Average daily $PM_{2.5}$ concentrations across the US during the study period (2001-2013)
Figure S5. Exposure-response relationship between prenatal $PM_{2.5}$ exposure and risk of autism spectrum disorder
Figure S6. Exposure-response relationship between postnatal $PM_{2.5}$ exposure and risk of developmental coordination disorder
Figure S7. Lag-response relationship between $PM_{2.5}$ concentration (10 μg/m^3 increase) and NDD during preconception and prenatal period (adjusted for temperature, relative humidity, individual and area-level covariates) at different lag times

Table S1. Definition of neurodevelopmental disorders

Outcome	Algorithm
Autism Spectrum Disorder	At ≥ 1 year of age: ≥ 2 dates with ICD-9 Dx 299.xx (except 299.1x)
Attention Deficit Hyperactivity Disorder	At ≥ 2 years of age, any of the following:<br>• ≥ 2 dates with ICD-9 Dx 314.xx<br>• ≥ 2 dispenses of atomoxetine, clonidine, guanfacine, (dextro/lisdex) amphetamine, (dex)methylphenidate<br>• ≥ 1 Dx 314.xx & ≥ 1 dispensing
Learning Difficulty	At ≥ 2 years of age: ≥ 1 ICD-9 Dx 315.0x, 315.1, 315.2
Developmental Speech or Language Disorder	At ≥ 1.5 years of age: ≥ 2 dates with ICD-9 Dx 315.3x (except 315.34)
Intellectual disability	At ≥ 2 years of age: ≥ 2 dates with ICD-9 Dx 317, 318.x, 319
Developmental Coordination Disorder	≥ 2 dates with ICD-9 Dx 315.4 (any age)
Behavioral Disorder	At ≥ 2 years of age: ≥ 2 dates with ICD-9 Dx 312.xx, 313.xx

Table S2. Definition of select individual and area-level covariates

Variable	Level	Coding
Substance abuse	Individual	Binary (yes/no). Whether mother had used opioids, cannabis, sedatives, cocaine, other stimulants, hallucinogens, nicotine, inhalant abuse, and other psychoactive drugs
Smoking	Individual	Binary (yes/no). Whether mother did tobacco smoking, chewing, or nicotine
Alcohol abuse	Individual	Binary (yes/no). Whether mother abused alcohol or had alcohol-related diseases (e.g., alcoholic fatty liver, alcohol-induced mental disorders).

Poor nutrition	Individual	Binary (yes/no). Whether mother was deficient in nutritional elements (e.g., vitamins, zinc, calcium, selenium).
Folate intake	Individual	Binary (yes/no). Whether mother had a prescription for folic acid during pregnancy
Normalized Difference Vegetation Index (NDVI)	Area-level	Numeric. An indicator of vegetation health and density quantified by the ratio of near-infrared to visible light, which ranges between values of -1 to 1 (1 signals high green space, values close to 0 represent no green space, and -1 are generally water bodies). NDVI data were obtained from Landsat 7 and Landsat 8, with images generated every 16 days at 30m resolution, aggregated by zip code and year, and linked to each participant's zip code.
Distance to nearest hospital	Area-level	Numeric. Quantifies the distance from the centroid of each zip code tabulation area to the nearest hospital of each individual's residential zip code using the Dartmouth Atlas of Health Care
Population density	Area-level	Numeric. ~1 km resolution obtained from NASA's Gridded Population of the World, aggregated by zip code and year, and linked to each participant's zip code.
Household income	Area-level	Numeric. Total income of all residents within a household who are 15+ years of age. This includes income from various sources like wages, salaries, and pensions.
Value of owner-occupied housing	Area-level	Numeric. An estimate of the selling price of the owner-occupied home
Percentage living below the poverty level	Area-level	Numeric. The poverty level is a federal measure of income set by the U.S. Census Bureau and adjusted annually for inflation using the Consumer Price Index (CPI). It varies by family size and composition. In 2021, the poverty threshold for a single person was $13,788 per year.
Percentage with less than high school education	Area-level	Numeric. Proportion of individuals whose highest education level is below a high school diploma or its equivalent (e.g. GED).
Percentage of owner-occupied housing units	Area-level	Numeric. Proportion of occupied housing units where the owner (or one of co-owners) lives in the unit (i.e. homeownership rate).

Table S3. Cohort characteristics stratified by neurodevelopmental disease diagnosis (n=1,547,244)

	**Total**	**ASD**	**ADHD**	**Any NDD**
Individual variables	n=1,547,244	n=12,942	n=67,338	n=162,150
Sex of child (Male) n (%)	781,358 (50.5)	9,965 (77.0)	45,991 (68.3)	105,560 (65.1)
Maternal race/ethnic group n (%)				
White	642,102 (41.5)	6,096 (47.1)	33,737 (50.1)	77,021 (47.5)
Black/African-American	502,854 (32.5)	3,223 (24.9)	24,515 (36.4)	51,393 (31.7)
Asian/Other Pacific	49,512 (3.2)	492 (3.8)	471 (0.7)	3,243 (2.0)
Hispanic/Latino	300,165 (19.4)	2,718 (21.0)	7,205 (10.7)	26,106 (16.1)
Unknown	51,059 (3.3)	414 (3.2)	1,414 (2.1)	4,216 (2.6)
Maternal age, mean (SD)	24.5 (5.9)	26.3 (6.2)	23.8 (5.4)	24.7 (5.8)
<18 n (%)	134,610 (8.7)	556 (4.3)	5,656 (8.4)	11,512 (7.1)
18–45 n (%)	1,411,534 (91.3)	12,170 (95.6)	61,209 (91.6)	150,475 (92.8)
>45 n (%)	1,100 (0.0)	200(0.0)	413 (0.0)	900(0.0)

Low birth weight n (%)	116,043 (7.5)	1,048 (8.1)	5,319 (7.9)	12,972 (8.0)
Gestational diabetes n (%)	137,704 (8.9)	1,631 (12.6)	6,195 (9.2)	16,376 (10.1)
Gestational hypertension n (%)	55,701 (3.6)	582 (4.5)	2,828 (4.2)	6,648 (4.1)
Substance abuse n (%)	20,114 (1.3)	233 (1.8)	1,212 (1.8)	2,919 (1.8)
Smoking n (%)	30,945 (2.0)	285 (2.2)	1,683 (2.5)	3,891 (2.4)
Alcohol abuse n (%)	7,736 (0.5)	65 (0.5)	471 (0.7)	973 (0.6)
Maternal mental health n (%)*	139,356 (9.0)	1,852 (14.3)	9,174 (13.6)	20,693 (12.8)
Poor nutrition n (%)	6,189 (0.4)	65 (0.5)	202 (0.3)	811 (0.5)
Prenatal folate n (%)	55,701 (3.6)	712 (5.5)	2,559 (3.8)	6,972 (4.3)
BMI, mean (SD)	27.7 (1.4)	27.6 (1.2)	27.8 (1.3)	27.8 (1.3)
Area-level variables				
Median household income ($1,000) [mean (sd)]	41.9 (15)	42.4 (15.2)	38.2 (12.8)	40.7 (14.3)
Median house value ($1,000) [mean (sd)]	180.8 (142.4)	216.0 (165.2)	131.5 (102.3)	171.2 (142.6)
Population density [mean (sd)]	7321 (15365.8)	12122.4 (21808.9)	4702.9 (12106.6)	7129.3 (15747.6)
nearest_hospital_km [mean (sd)]	5.5 (6.9)	5.0 (6.4)	5.8 (7.1)	5.6 (6.9)

*Includes any of the following: adjustment disorder, anxiety, alcohol abuse, bipolar disorder, depression, personality disorder, psychosis, schizophrenia, sleep disorders, and substance abuse.

Table S4. Hazard ratio for the association between average annual postnatal $PM_{2.5}$ exposure (10 μg/m^3 increase) and risk of neurodevelopmental disorders at cumulative lag (0-3 years), without adjustment for average prenatal and preconception $PM_{2.5}$ exposure and with adjustment average prenatal and preconception $PM_{2.5}$ exposure and prenatal and preconception $PM_{2.5}$ exposure as DLM

**Condition**	**Postnatal $PM_{2.5}$ exposure (not controlling for average prenatal and preconception exposure)**	**Postnatal $PM_{2.5}$ exposure (controlling for prenatal and preconception exposure as DLM)**
**Autism spectrum disorder**	1.15 [0.90, 1.47]	1.10 [0.75, 1.62]
**Attention deficit hyperactivity disorder**	0.87 [0.75, 1.00]	0.96 [0.81, 1.15]
**Learning difficulty**	0.81 [0.58, 1.12]	0.76 [0.49, 1.20]
**Developmental speech or language disorder**	0.81 [0.70, 0.94]	0.84 [0.69, 1.01]
**Developmental coordination disorder**	1.45 [0.81, 2.60]	1.81 [0.94, 3.47]
**Intellectual disability**	0.83 [0.52, 1.30]	1.17 [0.58, 2.36]
**Behavioral disorder**	1.00 [0.86, 1.16]	1.01 [0.91, 1.12]

Table S5. Hazard ratio [95% CI] for association between $PM_{2.5}$ exposure and NDD during each year of follow up in the postnatal period

Condition	Year 1	Year 2	Year 3
**Autism spectrum disorder**	1.33 [0.88, 2.02]	1.10 [0.78, 1.56]	0.86 [0.63, 1.17]
**Attention deficit hyperactivity disorder**	1.17 [0.94, 1.46]	0.90 [0.74, 1.09]	0.82 [0.69, 0.97]
**Learning difficulty**	1.03 [0.61, 1.74]	0.72 [0.44, 1.21]	0.72 [0.46, 1.11]
**Developmental speech or language disorder**	1.10 [0.89, 1.35]	1.05 [0.88, 1.26]	0.75 [0.64, 0.87]
**Developmental coordination disorder**	1.57 [0.77, 3.20]	1.72 [0.97, 3.07]	1.23 [0.78, 1.93]
**Intellectual disability**	1.91 [0.84, 4.36]	1.76 [0.88, 3.54]	0.62 [0.37, 1.05]
**Behavioral disorder**	0.92 [0.78, 1.08]	1.02 [0.84, 1.23]	1.09 [0.93, 1.28]

Table S6. Hazard ratios [95% CI] for association between average weekly preconception and prenatal $PM_{2.5}$ exposure (10 $\mu g/m^3$ increase) and risk of neurodevelopmental disorders at the cumulative lag (12 weeks preconception and 37 weeks prenatal) from models stratified by sex.

Condition	Preconception			Prenatal		
	**Overall**	**Male**	**Female**	**Overall**	**Male**	**Female**
**Autism spectrum disorder**	1.02 [0.94, 1.11]	1.01 [0.92, 1.10]	1.08 [0.91, 1.30]	1.17 [1.00, 1.37]	1.15 [0.97, 1.37]	1.28 [0.93, 1.76]
**Attention deficit hyperactivity disorder**	1.03 [0.99, 1.08]	1.04 [0.99, 1.09]	1.00 [0.93, 1.08]	0.90 [0.83, 0.99]	0.94 [0.85, 1.03]	0.82 [0.71, 0.95]
**Learning difficulty**	1.03 [0.94, 1.14]	1.03 [0.91, 1.17]	1.01 [0.86, 1.18]	0.92 [0.75, 1.13]	1.02 [0.78, 1.33]	0.73 [0.53, 0.99]
**Developmental speech or language disorder**	0.98 [0.94, 1.02]	0.97 [0.92, 1.02]	1.01 [0.95, 1.08]	0.96 [0.88, 1.05]	0.94 [0.85, 1.04]	0.99 [0.87, 1.13]
**Developmental coordination disorder**	1.05 [0.92, 1.20]	1.06 [0.91, 1.24]	1.03 [0.84, 1.27]	1.04 [0.76, 1.44]	1.11 [0.76, 1.61]	0.91 [0.56, 1.47]
**Intellectual disability**	0.88 [0.76, 1.02]	0.89 [0.74, 1.07]	0.90 [0.71, 1.14]	0.93 [0.70, 1.24]	0.87 [0.62, 1.22]	1.11 [0.68, 1.81]
**Behavioral disorder**	0.98 [0.94, 1.01]	0.96 [0.92, 1.01]	1.01 [0.94, 1.08]	1.03 [0.95, 1.11]	1.04 [0.94, 1.15]	0.99 [0.87, 1.12]

Table S7. Hazard ratios [95% CI] for association between average weekly preconception and prenatal $PM_{2.5}$ exposure (10 $\mu g/m^3$ increase) and risk of neurodevelopmental disorders at the cumulative lag from models stratified by urbanicity (> or < 500 residents per zip code)

Condition	Preconception			Prenatal		
	**Urban (>500 people/zip code)**	**Suburban/Rural (<500 people/zip code)**	**Overall**	**Urban (>500 people/zip code)**	**Suburban/Rural (<500 people/zip code)**	**Overall**
Autism spectrum disorder	1.08 [0.97, 1.21]	0.99 [0.87,1.14]	1.02 [0.94, 1.11]	1.35 [1.06, 1.72]	1.11 [0.85, 1.46]	1.17 [1.00, 1.37]
Attention deficit hyperactivity disorder	1.01 [0.95, 1.06]	1.06 [0.99, 1.13]	1.03 [0.99, 1.08]	0.77 [0.67, 0.88]	1.04 [0.91, 1.20]	0.90 [0.83, 0.99]
Learning difficulty	1.01 [0.88, 1.16]	1.05 [0.89, 1.24]	1.03 [0.94, 1.14]	0.92 [0.68, 1.26]	0.85 [0.61, 1.20]	0.92 [0.75, 1.13]

Developmental speech or language disorder	0.97 [0.92,1.02]	1.00 [0.94, 1.07]	0.98 [0.94, 1.02]	0.94 [0.85, 1.04]	0.98 [0.85, 1.15]	0.96 [0.88, 1.05]
Developmental coordination disorder	1.06 [0.86, 1.27]	1.01 [0.81, 1.25]	1.05 [0.92, 1.20]	1.31 [0.82, 2.07]	0.75 [0.47, 1.19]	1.04 [0.76, 1.44]
Intellectual disability	0.97 [0.79, 1.19]	0.75 [0.58, 0.97]	0.88 [0.76, 1.02]	0.89 [0.51, 1.22]	0.95 [0.60, 1.40]	0.93 [0.70, 1.24]
Behavioral disorder	N/A	1.03 [0.97, 1.10]	0.98 [0.94, 1.01]	N/A	1.23 [1.07, 1.40]	1.03 [0.95, 1.11]

N/A=not applicable; model didn't converge (low sample size)

Table S8. Hazard ratios [95% CI] for association between average weekly preconception and prenatal $PM_{2.5}$ exposure (10 μg/m^3 increase) and risk of neurodevelopmental disorders at the cumulative lag from models stratified by US region

**Preconception**						
**Condition**	**Overall**	**Northeast**	**Southeast**	**Midwest**	**West**	**Southwest**
**Autism spectrum disorder**	1.02 [0.94, 1.11]	0.91 [0.70, 1.18]	1.03 [0.76, 1.40]	1.02 [0.81, 1.28]	0.98 [0.87, 1.11]	1.16 [0.45, 3.00]
**Attention deficit hyperactivity disorder**	1.03 [0.99, 1.08]	1.11 [0.79, 1.56]	1.10 [1.00, 1.22]	0.99 [0.90, 1.09]	0.97 [0.90, 1.03]	0.91 [0.59, 1.38]
**Learning difficulty**	1.03 [0.94, 1.14]	1.08 [0.77, 1.52]	0.92 [0.62, 1.35]	1.03 [0.77, 1.39]	1.02 [0.87, 1.19]	0.37 [0.16, 0.87]
**Developmental speech or language disorder**	0.98 [0.94, 1.02]	1.00 [0.89, 1.12]	0.90 [0.81, 1.01]	1.01 [0.92, 1.10]	0.96 [0.89, 1.02]	1.18 [0.86, 1.61]
**Developmental coordination disorder**	1.05 [0.92, 1.20]	1.01 [0.79, 1.30]	0.72 [0.49, 1.07]	1.21 [0.89, 1.64]	1.09 [0.82, 1.44]	0.85 [0.38, 1.91]
**Intellectual disability**	0.88 [0.76, 1.02]	0.62 [0.38, 1.00]	0.74 [0.45, 1.22]	0.90 [0.60, 1.36]	0.85 [0.68, 1.07]	2.53 [0.18, 36.24]
**Behavioral disorder**	0.98 [0.94, 1.01]	1.20 [0.86, 1.69]	0.97 [0.85, 1.11]	0.97 [0.87, 1.09]	0.99 [0.94, 1.05]	1.04 [0.66, 1.65]
**Prenatal**						
**Condition**	**Overall**	**Northeast**	**Southeast**	**Midwest**	**West**	**Southwest**
**Autism spectrum disorder**	1.17 [1.00, 1.37]	1.41 [0.79, 2.52]	1.42 [0.74, 2.76]	1.11 [0.64, 1.94]	1.21 [0.99, 1.47]	1.50 [0.28, 8.05]
**Attention deficit hyperactivity disorder**	0.90 [0.83, 0.99]	0.42 [0.20, 0.89]	0.82 [0.65, 1.05]	1.01 [0.81, 1.25]	0.91 [0.90, 1.05]	0.99 [0.47, 2.09]
**Learning difficulty**	0.92 [0.75, 1.13]	0.72 [0.34, 1.52]	1.04 [0.43, 2.50]	1.15 [0.61, 2.15]	0.86 [0.65, 1.15]	0.16 [0.02, 1.02]
**Developmental speech or language disorder**	0.96 [0.88, 1.05]	1.23 [0.96, 1.59]	0.79 [0.62, 1.01]	1.14 [0.94, 1.39]	0.87 [0.76, 1.00]	1.36 [0.83, 2.21]
**Developmental coordination disorder**	1.04 [0.76, 1.44]	1.75 [0.97, 3.18]	0.66 [0.29, 1.50]	0.97 [0.49, 1.90]	1.16 [0.58, 2.32]	0.67 [0.17, 2.73]
**Intellectual disability**	0.93 [0.70, 1.24]	0.78 [0.24, 2.48]	1.21 [0.44, 3.36]	0.64 [0.25, 1.64]	1.01 [0.72, 1.42]	0.16 [0.01, 9.10]
**Behavioral disorder**	1.03 [0.95, 1.11]	0.96 [0.80, 1.14]	1.24 [0.95, 1.62]	1.34 [1.05, 1.71]	0.99 [0.89, 1.11]	1.52 [0.68, 3.44]

Table S9. Hazard ratios [95% CI] for association between average annual postnatal $PM_{2.5}$ exposure (10 μg/m^3 increase) and risk of neurodevelopmental disorders (controlling for prenatal and preconception $PM_{2.5}$ exposure) at cumulative lag (0-3 years) from models stratified by sex

**Condition**	**Male**	**Female**	**Overall**
**Autism spectrum disorder**	1.07 [0.73, 1.58]	1.58 [0.76, 3.28]	1.16 [0.79, 1.70]

**Attention deficit hyperactivity disorder**	1.12 [0.91, 1.37]	1.06 [0.78, 1.43]	1.10 [0.92, 1.31]
**Learning difficulty**	0.92 [0.52, 1.64]	0.52 [0.26, 1.03]	0.77 [0.51, 1.16]
**Developmental speech or language disorder**	0.86 [0.70, 1.06]	0.76 [0.56, 1.02]	0.82 [0.68, 0.98]
**Developmental coordination disorder**	1.50 [0.69, 3.28]	2.38 [0.92, 6.11]	1.77 [0.93, 3.34]
**Intellectual disability**	1.11 [0.52, 2.34]	1.76 [0.56, 5.49]	1.16 [0.59, 2.28]
**Behavioral disorder**	0.96 [0.77, 1.19]	1.02 [0.76, 1.36]	0.97 [0.81, 1.17]

Table S10. Hazard ratios [95% CI] for association between average annual postnatal $PM_{2.5}$ exposure (10 $\mu g/m^3$ increase) and risk of neurodevelopmental disorders (controlling for prenatal and preconception $PM_{2.5}$ exposure) at cumulative lag (0-3 years) from models stratified by urbanicity (> or < 500 residents per zip code)

Condition	Urban (>500 people/zip code)	Suburban/Rural (<500 people/zip code)	Overall
**Autism spectrum disorder**	1.57 [0.96, 2.55]	1.12 [0.67, 1.87]	1.16 [0.79, 1.70]
**Attention deficit hyperactivity disorder**	1.07 [0.83, 1.39]	1.41 [1.09, 1.81]	1.10 [0.92, 1.31]
**Learning difficulty**	0.65 [0.35, 1.20]	0.76 [0.38, 1.51]	0.71 [0.52, 1.16]
**Developmental speech or language disorder**	0.88 [0.67, 1.15]	0.78 [0.59, 1.03]	0.82 [0.68, 0.98]
**Developmental coordination disorder**	4.96 [1.98, 12.48]	0.56 [0.23, 1.37]	1.77 [0.93, 3.40]
**Intellectual disability**	1.17 [0.39, 3.50]	0.76 [0.27, 2.13]	1.16 [0.59, 2.28]
**Behavioral disorder**	0.86 [0.66, 1.13]	1.26 [0.94, 1.68]	0.97 [0.81, 1.17]

Table S11. Hazard ratios [95% CI] for association between average weekly preconception and prenatal $PM_{2.5}$ exposure (10 $\mu g/m^3$ increase) and risk of neurodevelopmental disorders at the cumulative lag (12 weeks preconception and 37 weeks prenatal) at different lag times

Condition	37 weeks	30 weeks	26 weeks
Autism spectrum disorder	1.19 [0.98, 1.45]	1.15 [0.97, 1.37]	1.05 [0.89, 1.22]
Attention deficit hyperactivity disorder	0.93 [0.83, 1.04]	0.94 [0.86, 1.04]	1.01 [0.92, 1.10]
Learning difficulty	0.95 [0.75, 1.22]	1.04 [0.84, 1.28]	1.05 [0.87, 1.28]
Developmental speech or language disorder	0.93 [0.84, 1.04]	0.95 [0.87, 1.04]	0.96 [0.88, 1.05]
Developmental coordination disorder	1.10 [0.73, 1.64]	1.27 [0.89, 1.80]	1.24 [0.91, 1.69]
Intellectual disability	0.82 [0.57, 1.18]	0.82 [0.59, 1.14]	0.85 [0.63, 1.15]
Behavioral disorder	1.00 [0.91, 1.11]	1.00 [0.92, 1.09]	1.00 [0.93, 1.08]

Table S12. Hazard ratios [95% CI] for association between average weekly preconception and prenatal $PM_{2.5}$ exposure (10 μg/m^3 increase) and risk of neurodevelopmental disorders among children diagnosed at age 3 years or later

Condition	Preconception and prenatal $PM_{2.5}$ exposure		Postnatal $PM_{2.5}$ exposure	
	All diagnoses	Diagnosed at age 3 or later	All diagnoses	Diagnosed at age 3 or later
Autism spectrum disorder	1.19 [0.98, 1.45]	1.15 [0.92, 1.44]	1.16 [0.79, 1.70]	1.28 [0.83, 1.97]
Attention deficit hyperactivity disorder	0.93 [0.83, 1.04]	0.98 [0.88, 1.09]	1.10 [0.92, 1.31]	1.00 [0.83, 1.20]
Learning difficulty	0.95 [0.75, 1.22]	0.90 [0.69, 1.16]	0.77 [0.52, 1.16]	0.72 [0.46, 1.14]
Developmental speech or language disorder	0.93 [0.84, 1.04]	0.87 [0.75, 1.00]	0.82 [0.68, 0.98]	0.73 [0.56, 0.96]
Developmental coordination disorder	1.10 [0.73, 1.64]	1.31 [0.71, 2.43]	1.77 [0.93, 3.40]	0.84 [0.28, 2.55]
Intellectual disability	0.82 [0.57, 1.18]	0.82 [0.55, 1.21]	1.16 [0.59, 2.28]	1.12 [0.50, 2.51]
Behavioral disorder	1.00 [0.91, 1.11]	1.03 [0.93, 1.15]	0.97 [0.81, 1.17]	1.01 [0.82, 1.25]

Table S13. Hazard ratios [95% CI] for association between average weekly preconception and prenatal $PM_{2.5}$ exposure (10 μg/m^3 increase) and risk of neurodevelopmental disorders at the cumulative lag (12 weeks preconception and 37 weeks prenatal and 0-3 years postnatal), controlling for average cumulative NO_2 concentration

Condition	Preconception and prenatal $PM_{2.5}$ exposure		Postnatal $PM_{2.5}$ exposure	
	Not controlling for NO_2 concentration	Controlling for NO_2 concentration	Not controlling for NO_2 concentration	Controlling for NO_2 concentration
Autism spectrum disorder	1.19 [0.98, 1.45]	1.13 [0.91, 1.41]	1.16 [0.79, 1.70]	0.98 [0.71, 1.36]
Attention deficit hyperactivity disorder	0.93 [0.83, 1.04]	0.99 [0.88, 1.11]	1.10 [0.92, 1.31]	1.15 [0.95, 1.39]
Learning difficulty	0.95 [0.75, 1.22]	0.90 [0.69, 1.18]	0.77 [0.52, 1.16]	0.65 [0.40, 1.05]
Developmental speech or language disorder	0.93 [0.84, 1.04]	0.93 [0.83, 1.04]	0.82 [0.68, 0.98]	0.85 [0.70, 1.03]
Developmental coordination disorder	1.10 [0.73, 1.64]	1.15 [0.77, 1.72]	1.77 [0.93, 3.40]	1.90 [0.97, 3.73]
Intellectual disability	0.82 [0.57, 1.18]	0.68 [0.45, 1.02]	1.16 [0.59, 2.28]	1.11 [0.54, 2.26]
Behavioral disorder	1.00 [0.91, 1.11]	1.00 [0.90, 1.11]	0.97 [0.81, 1.17]	0.98 [0.80, 1.20]

Table S14. Hazard ratios [95% CI] for the association between average preconception, prenatal and postnatal $PM_{2.5}$ exposure (10 μg/m^3 increase) and risk of neurodevelopmental disorders, accounting for inverse probability of censoring weights

	Preconception	Prenatal	Postnatal
ASD			
adjusted main analysis	1.00 (0.90-1.09)	1.08 (1,01-1.16)	1.13 (0.85-1.42)
accounting for censoring weights	0.98(0.89-1.11)	1.06 (1.00-1.18)	1.10 (0.88-1.47)
ADHD			
adjusted main analysis	1.01 (0.99-1.10)	0.95 (0.80-1.10)	1.10 (0.90-1.27)
accounting for censoring weights	0.98 (0.93-1.15)	0.99 (0.85-1.12)	1.11 (0.92-1.30)
Learning difficulty			
adjusted main analysis	1.01 (0.90 -1.09)	1.00 (0.72-1.21)	0.73 (0.50-1.20)
accounting for censoring weights	1.00 (0.93-1.11)	0.98 (0.85-1.18)	0.72 (0.52-1.18)
Developmental speech or language disorder			
adjusted main analysis	1.01 (0.90-1.20)	1.00 (0.80-1.18)	0.80 (0.65-1.08)
accounting for censoring weights	1.03 (0.93-1.22)	1.00 (0.81-1.18)	0.83 (0.70-1.10)
Developmental Coordination Disorder			
adjusted main analysis	1.03 (0.90-1.25)	1.02 (0.88-1.23)	1.32 (0.92-2,00)
accounting for censoring weights	1.02 (0.93-1.28)	1.01 (0.90-1.20)	1.30 (0.94-2.04)
Intellectual disability			
adjusted main analysis	0.84 (0.74-1.10)	1.00 (0.75-1.25)	1.08 (0.63-2.00)
accounting for censoring weights	0.82 (0.75-1.09)	1.02 (0.73-1.27)	1.06 (0.60-1.98)
Behavioral disorder			
adjusted main analysis	1.00 (0.95-1.10)	1.05 (0.95-1.18)	1.00 (0.70-1.65)
accounting for censoring weights	0.99 (0.90-1.12)	1.02 (0.92-1.20)	0.99 (0.81-1.40)

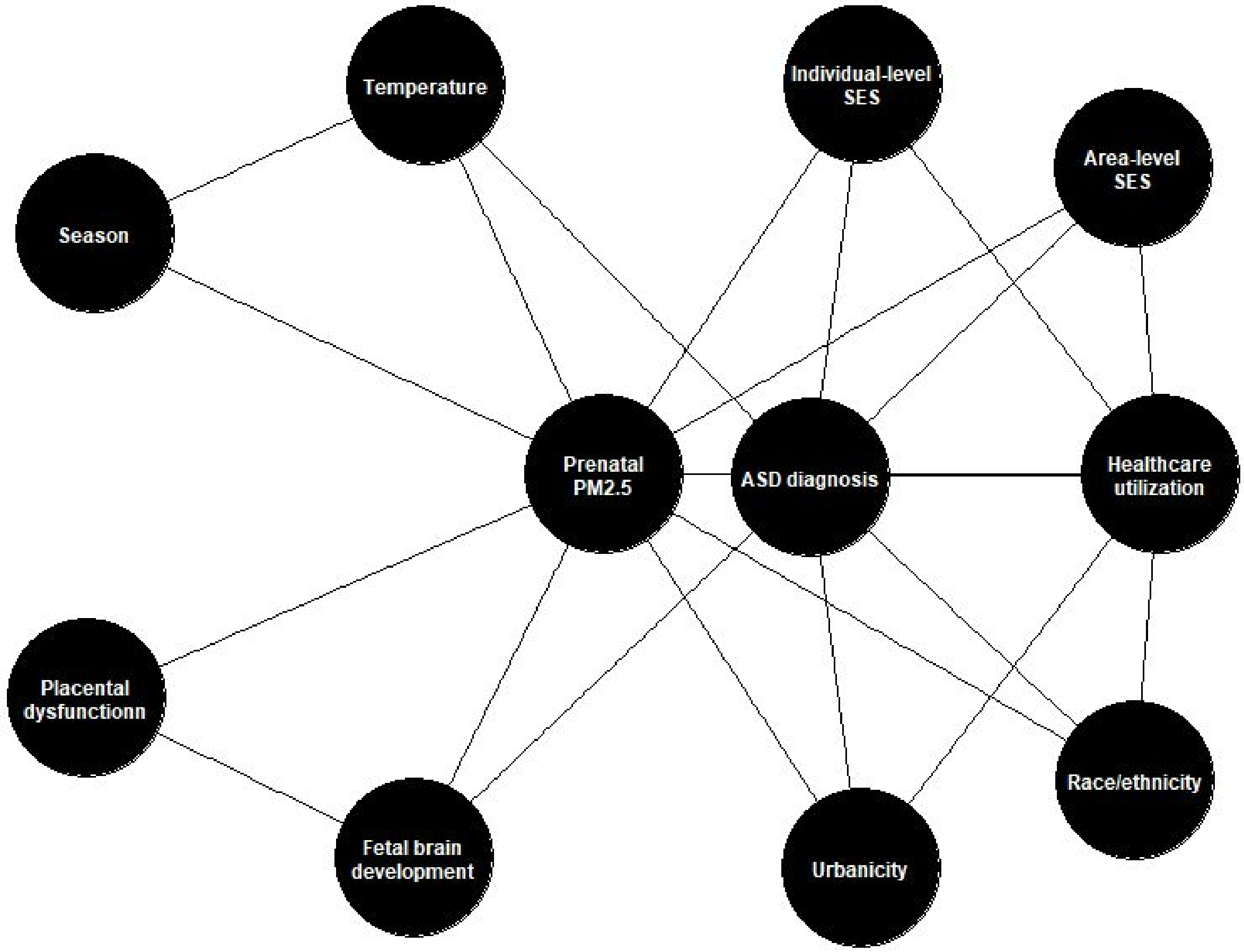

Figure S1. Direct acyclic graph of the association between prenatal $PM_{2.5}$ exposure and risk of autism spectrum disorder (ASD)

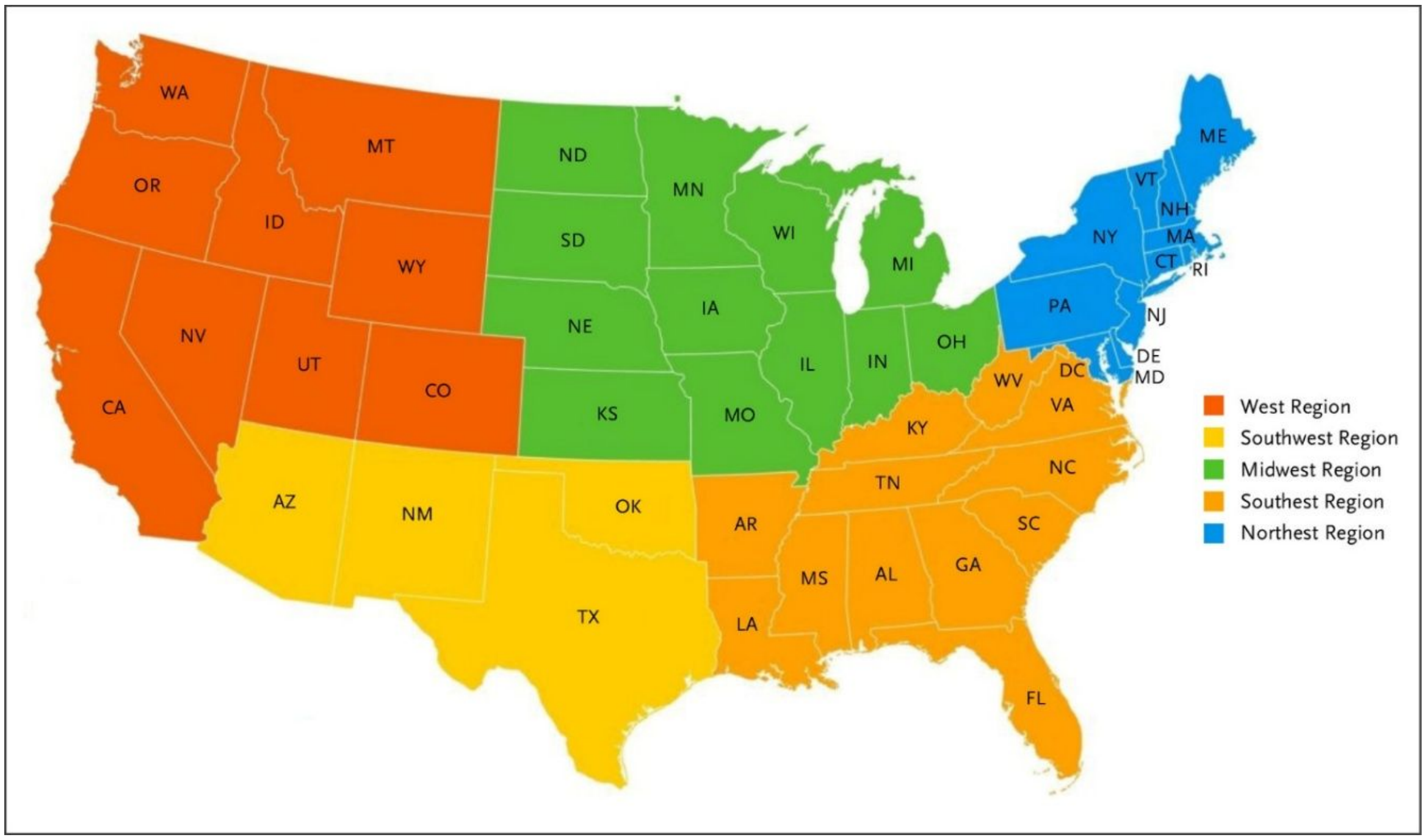

Figure S2. Map of five regions of the United States according to the US Census

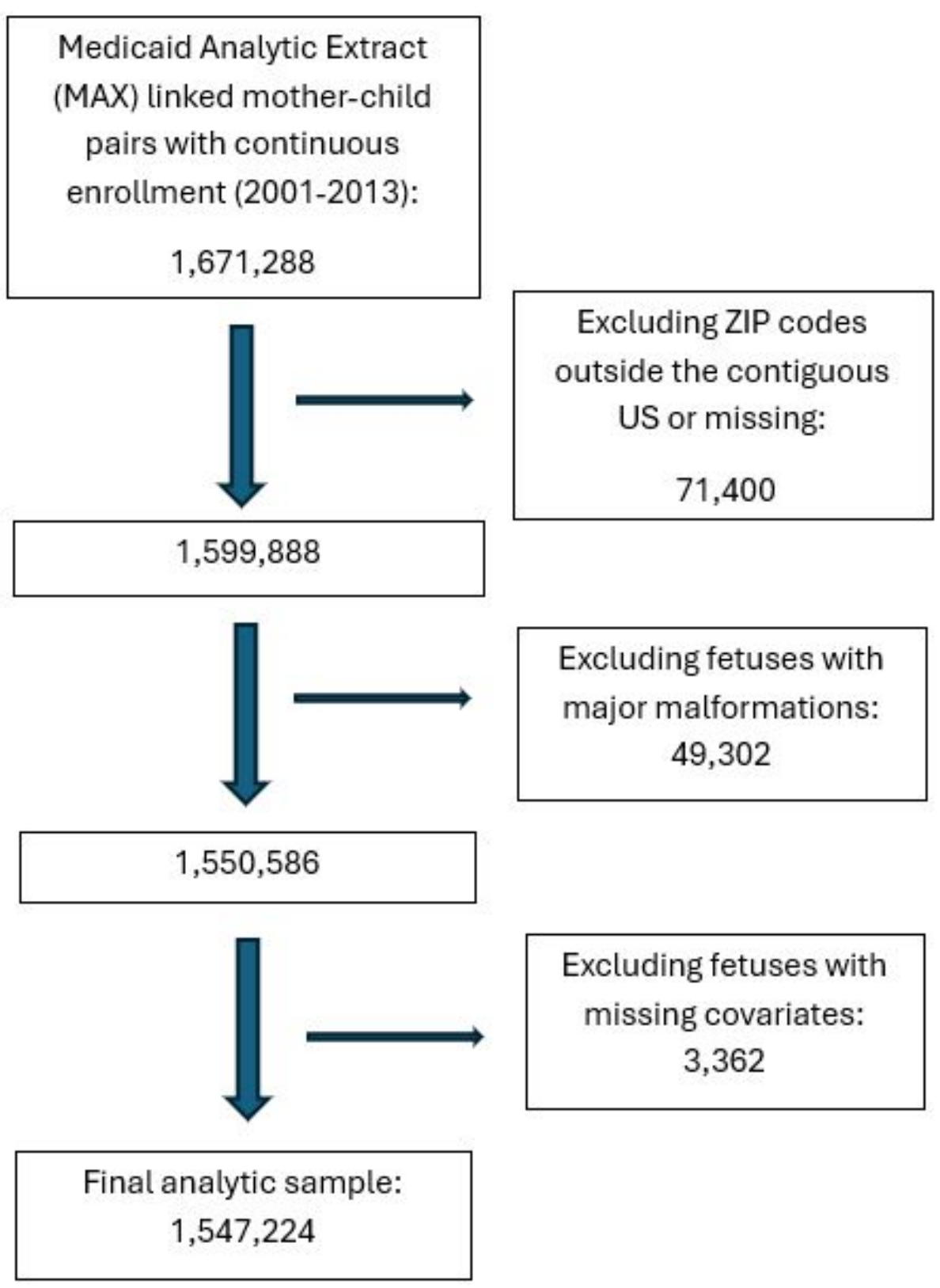

Figure S3. Flowchart describing arrival to the final analytic sample in the Medicaid Analytic eXtract database

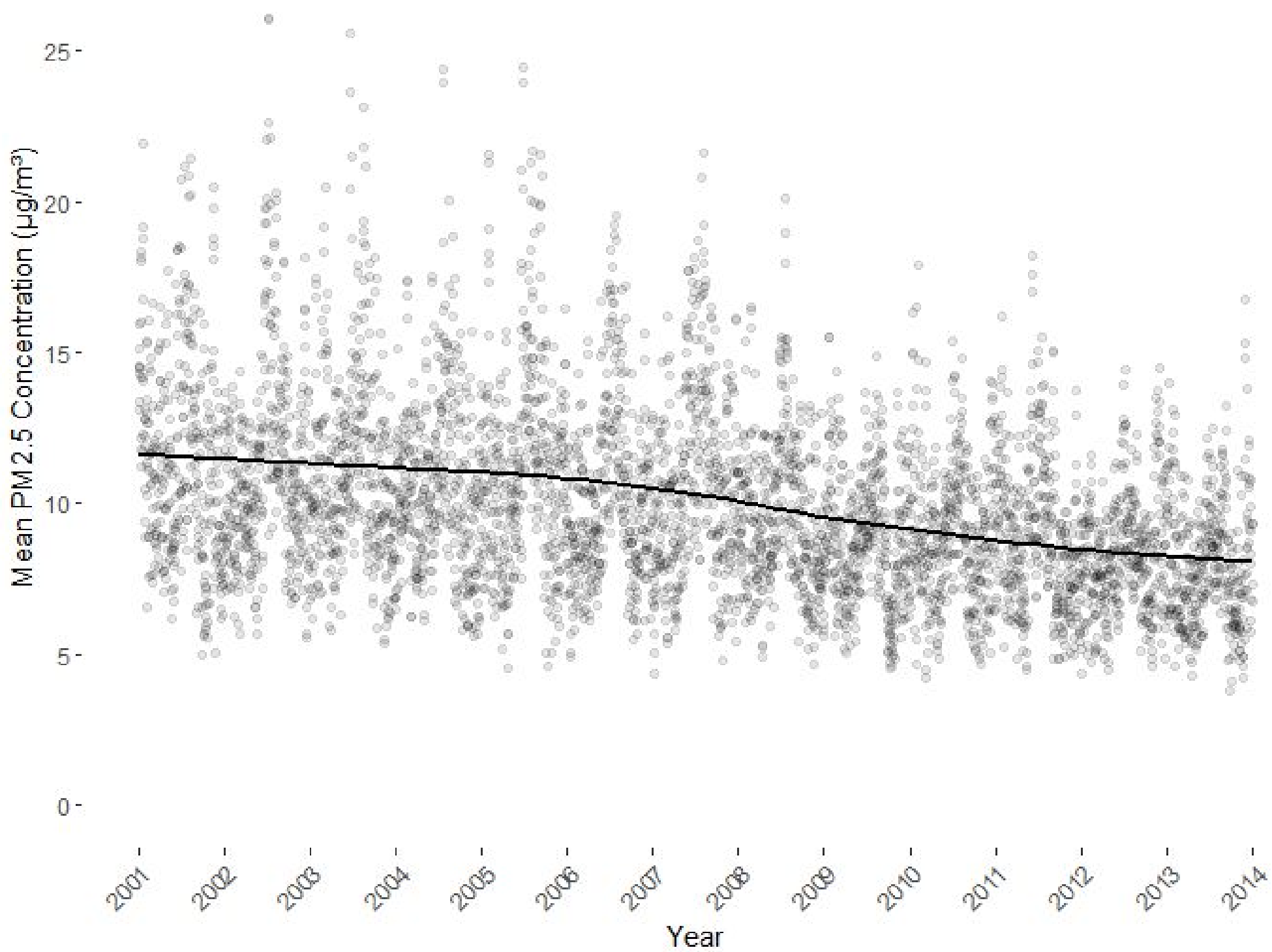

Figure S4. Average daily $PM_{2.5}$ concentrations across the US during the study period (2001-2013)

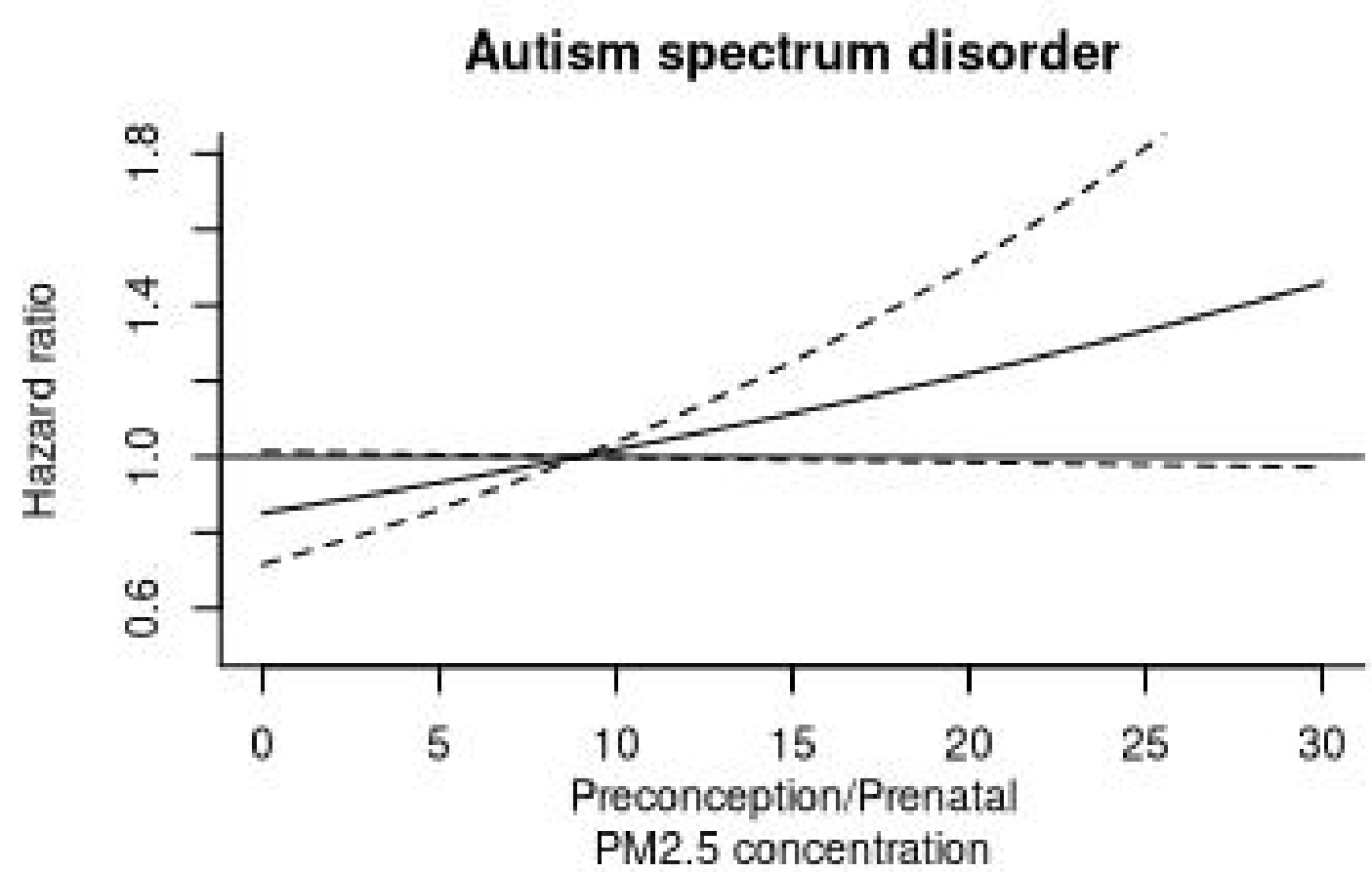

Figure S5. Exposure-response relationship between preconception and prenatal $PM_{2.5}$ exposure and risk of autism spectrum disorder

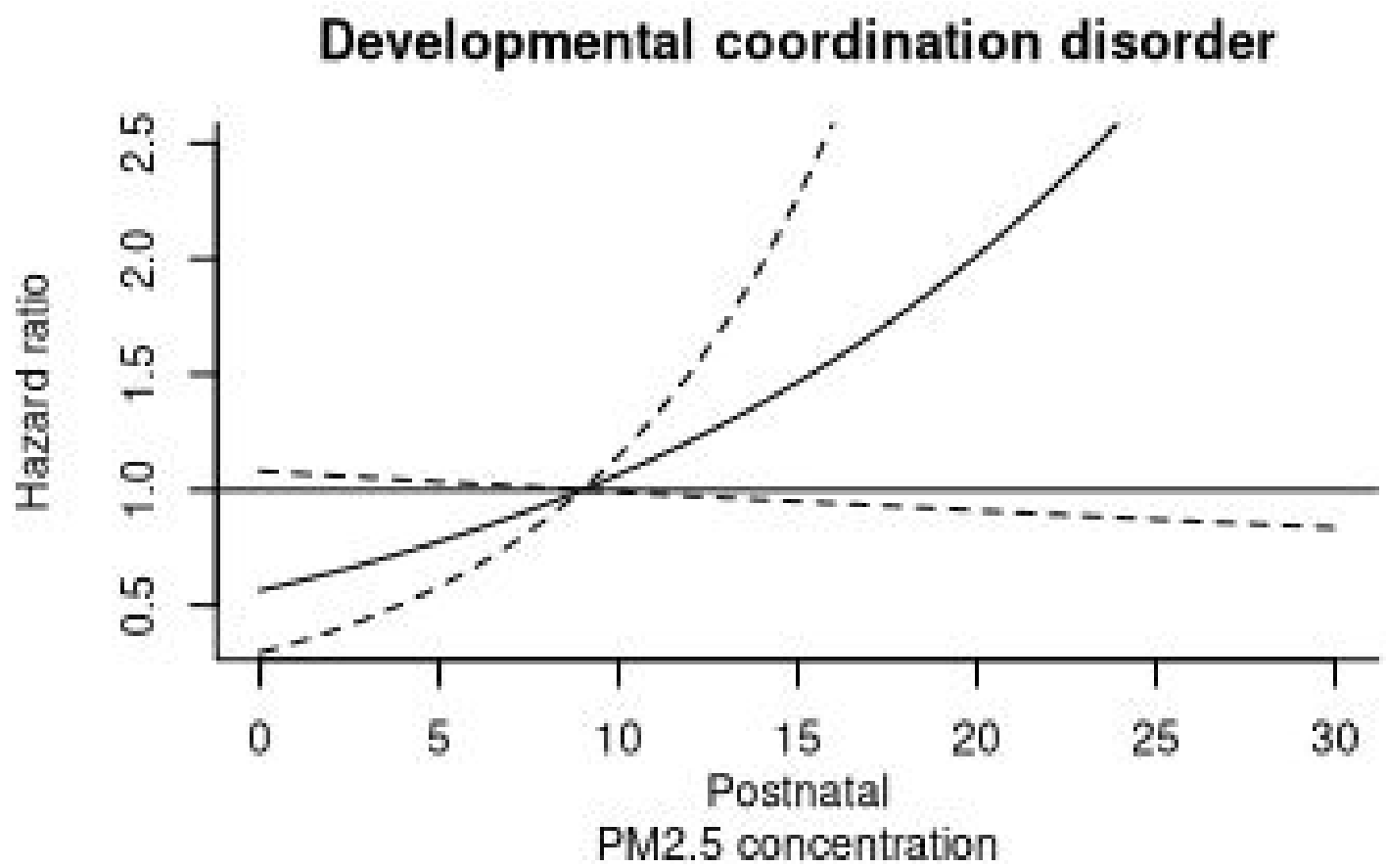

Figure S6. Exposure-response relationship between postnatal $PM_{2.5}$ exposure and risk of developmental coordination disorder

Figure S7. Lag-response relationship between $PM_{2.5}$ concentration (10 μg/m^3 increase) and NDD during preconception and prenatal period (adjusted for temperature, relative humidity, individual and area-level covariates) at different lag times.

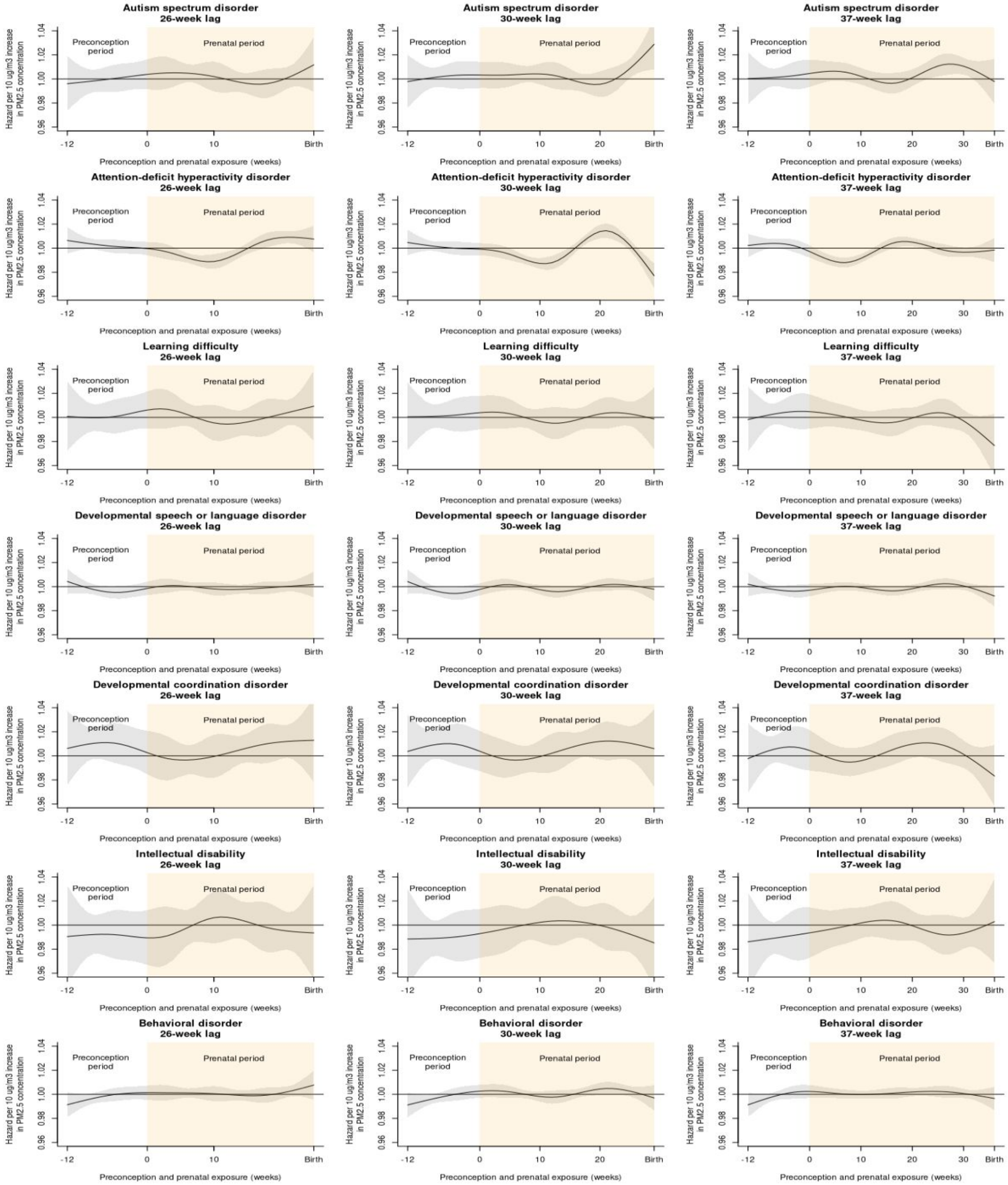

Supplement: Supplementary file 1 [file es6c03766_si_001.pdf]
